# Polypropylene-Rendered Antiviral by Three-Dimensionally Surface-Grafted Poly(*N*-benzyl-4-vinylpyridinium bromide)

**DOI:** 10.1021/acsami.3c15125

**Published:** 2024-02-12

**Authors:** Rie Hirao, Hisato Takeuchi, Jumpei Kawada, Nobuhiro Ishida

**Affiliations:** † Toyota Central R&D Labs, Inc., Nagakute, Aichi 480-1192, Japan

**Keywords:** polypropylene, antiviral, covalent bond, quaternary ammonium compounds, graft polymerization

## Abstract

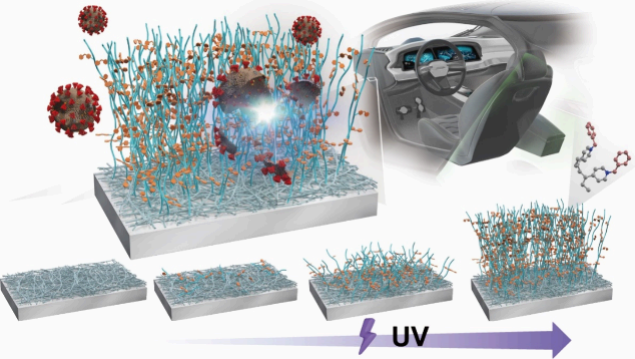

To inhibit viral infection, it is necessary for the surface of polypropylene (PP), a polymer of significant industrial relevance, to possess biocidal properties. However, due to its low surface energy, PP weakly interacts with other organic molecules. The biocidal effects of quaternary ammonium compounds (QACs) have inspired the development of nonwoven PP fibers with surface-bound quaternary ammonium (QA). Despite this advancement, there is limited knowledge regarding the durability of these coatings against scratching and abrasion. It is hypothesized that the durability could be improved if the thickness of the coating layer were controlled and increased. We herein functionalized PP with three-dimensionally surface-grafted poly(*N*-benzyl-4-vinylpyridinium bromide) (PBVP) by a simple and rapid method involving graft polymerization and benzylation and examined the influence of different factors on the antiviral effect of the resulting plastic by using a plaque assay. The thickness of the PBVP coating, surface roughness, and amount of QACs, which jointly determine biocidal activity, could be controlled by adjusting the duration and intensity of the ultraviolet irradiation used for grafting. The best-performing sample reduced the viral infection titer of an enveloped model virus (bacteriophage ϕ6) by approximately 5 orders of magnitude after 60 min of contact and retained its antiviral activity after surface polishing-simulated scratching and abrasion, which indicated the localization of QACs across the coating interior. Our method may expand the scope of application to resin plates as well as fibers of PP. Given that the developed approach is not limited to PP and may be applied to other low-surface-energy olefinic polymers such as polyethylene and polybutene, our work paves the way for the fabrication of a wide range of biocidal surfaces for use in diverse environments, helping to prevent viral infection.

## Introduction

Viral infectious disease outbreaks such as coronavirus disease 2019 (COVID-19), influenza, and Middle East respiratory syndrome have repeatedly occurred and may also occur in the future.^1−3^ The spread of such diseases is facilitated by the ability of viruses adhering to material surfaces to remain active for a certain period; for example, severe acute respiratory syndrome coronavirus 2 retains infectivity on metal, plastic, cotton, and surgical mask surfaces for periods from tens of hours to 7 days.^4−6^ Consequently, considerable attention has been drawn to the disinfection of material surfaces, which is typically accomplished with the help of chemicals, such as ethanol and surfactants. However, these agents have a low persistence on the material surface and must be reapplied on re-exposure to the virus. Other concerns raised in the wake of the massive disinfectant consumption during the pandemic are related to the environmental impact and disposal toxicity of disinfectants.^7,8^ The importance of hygienic environments necessitates the development of various antimicrobial and antiviral materials to meet the needs of diverse applications and has inspired the emergence of design methods based on both inorganic and organic materials.^7,9^ The inorganic materials used to impart biocidal activity when applied as coatings include silver,^10^ copper oxide,^11^ zinc oxide,^12,13^ and photocatalyst^14^ nanoparticles, which are easy to surface-process into resins and other materials but suffer from performance degradation due to oxidation and friction. Organic molecules with disinfectant properties include ethanol,^15^ surfactants,^16^ and quaternary ammonium compounds (QACs)^17−20^ and typically exhibit high solubility, which complicates the realization of a long-lasting effect on material surfaces. Fast, easy methods for applying long-lasting coatings have been reported, including simply coating from organic solvents^21^ and *in situ* copolymerization with self-assembling biocides.^22^ Antiviral materials have been less researched than antimicrobial ones but are not less important; moreover, designing materials specifically for viruses is essential because viruses strongly differ from bacteria in size, structure, and growth mechanism.^23−25^ The COVID-19 pandemic has further highlighted the importance of antiviral materials and inspired extensive research in this field,^7,9,26,27^ as exemplified by our previous works on surfactant-loaded mesoporous silica and nanopillar copper films.^28,29^ However, surfaces with long-lasting antiviral effects remain challenging to realize.

Polypropylene (PP) is a major industrially relevant polymer because of its high chemical stability, low density, strength, durability, and low cost. It is used to produce automotive parts, textiles, electrical products, medical equipment, and packaging.^30−32^ However, owing to its hydrocarbon chain-based molecular structure, PP exhibits low polarity and surface energy, weakly interacts with other organic molecules, and reluctantly engages in adhesion and bonding.^33^ Physicochemical surface treatments such as plasma polymerization,^34^ chemical grafting,^35,36^ and layer-by-layer deposition^37,38^ can increase the surface energy of PP and thus promote its interactions with other molecules and improve adhesion and coating properties. However, attempts to directly covalently bond ethanol or QACs to the PP surface have generally been unsuccessful because of the low reactivity of the PP surface. Therefore, the design of suitable antimicrobial and antiviral agents for the PP surface and their binding thereon are of key importance for realizing self-disinfecting PP surfaces.

Several innovative studies have attempted to endow PP with antibacterial and antiviral properties through surface treatment. In particular, numerous macromolecules with antimicrobial and antiviral activities have been developed owing to the dramatic progress in synthetic polymer chemistry.^39−41^ Regarding studies aimed at the immobilization of chemical agents on the PP surfaces, the discovery that soluble poly(vinylpyridinium halides) and poly(*N*-benzyl-4-vinylpyridinium salts) cross-linked to resin particles exhibit antibacterial activity has opened the possibility of binding antimicrobial molecules to polymer surfaces.^42−44^ Subsequently, the binding of such molecules to polyolefin surfaces was successfully achieved by forming a thin silica layer via combustion chemical vapor deposition followed by treatment with a silane coupling agent, acroylation, and copolymerization with vinylpyridine.^45,46^ A simpler approach has also been reported, namely, the synthesis of antimicrobial copolymers of hydrophobic *N*-alkyl- and benzophenone-containing poly(ethylenimine) from linear poly(2-ethyl-2-oxazoline) and the binding of this polymer to the PP surface via photo-cross-linking.^47^ These materials showed sufficient antimicrobial activity against Gram-positive and Gram-negative bacteria but were not tested against viruses at the time of their development. A PP nonwoven mask with ultraviolet (UV)-cross-linked dip- or spray-coated lignin with structural stability added with photosensitive methacrylate-containing QAC was reported,^48^ and the durability maintained its antiviral effect after 7 days of storage at 37 °C and 97% relative humidity. Quaternary ammonium (QA)-containing polymers were obtained by binding benzophenone substituted with C_12_-quaternary ammonium salts onto the fiber surface of melt-blown and spun-bonded PP nonwoven fabrics using UV irradiation^49^ and found to inactivate two types of enveloped viruses without interfering with the breathability of N95 respirator face masks. PP is a thermoplastic polymer with a wide range of applications; therefore, these technologies are expected to be deployed in more sanitary products. However, there is limited understanding regarding the durability of materials with antibacterial and antiviral agents bound to PP. For nonwoven fabrics made of PP, an ultrathin coating is essential to maintain breathability. In the context of PP plate resins, an ultrathin coating may peel off due to scratching and abrasion, decreasing antiviral properties. It is suggested that the durability could be improved if there were a method to control and increase the thickness of the coating layer.

**Figure 1:**
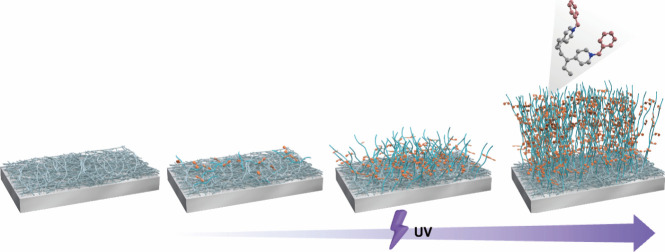
Schematic synthesis of poly(*N*-benzyl-4-vinylpyridinium bromide) (PBVP)-coated polypropylene (PP). The amount of quaternary ammonium compounds on the PP surface is determined by the time and intensity of ultraviolet irradiation used for the graft polymerization of PBVP.

Here, we propose an antiviral material in which PP is modified via the surface binding of a QAC-containing macromolecule, namely, poly(*N*-benzyl-4-vinylpyridinium bromide) (PBVP), and the surface properties and antiviral activity of the resulting material are examined as functions of fabrication conditions. The surface-bound PBVP chains are shown to elongate with increasing reaction time to form a three-dimensional (3D) structure (Figure 1), which endows PP with durable antiviral activity that is only marginally affected by abrasion and scuffing commonly occurring with commodity polymers. Whereas conventional synthetic methods require multiple steps and are limited in the amount of QACs that can be bound,^49^ our approach is simpler, enables the amount of QACs to be adjusted by controlling the photopolymerization conditions, and can potentially be applied to both filters and plate resins.

## Experimental Section

### Materials

PP (thickness of 1 mm) was purchased from AS ONE (Osaka, Japan). Benzophenone, dimethylformamide, benzyl bromide, and 4-vinylpyridine were purchased from Tokyo Kasei Kogyo (Tokyo, Japan). All other reagents were purchased from Fujifilm Wako Pure Chemical (Osaka, Japan).

### Synthesis

A piece of PP (10 mm × 10 mm) precleaned with acetone. The thus-precleaned sample was drop-coated with a solution of benzophenone (0.2 g, 1.1 mmol) in 4-vinylpyridine (1.0 g, 9.5 mmol), covered with a borosilicate cover glass and irradiated with UV light at λ = 365 nm using a light-emitting-diode device (CCS, Kyoto, Japan), and placed in a stainless-steel vat. The vat was covered with a glass plate and purged with nitrogen for 5 min. The specimen was then irradiated with intensities of 30–170 mW cm^–2^ for 1–20 min. After irradiation, the sample was sequentially washed with methanol, acetone, chloroform, and ethyl acetate and then sonicated in methanol for 10 min. Further, the sample was exposed to a solution of benzyl bromide (4.4 g, 25.7 mmol) in dimethylformamide (100 mL) and heated at 90 °C for 0.5–5 h under nitrogen upon slow stirring to form QACs on the grafted polymer chains. Finally, the sample was sequentially washed with methanol and acetone, sonicated in methanol for 10 min, vacuum-dried at 60 °C for 1 h, dipped in sterilized ultrapure water for 30 min, and air-dried at approximately 25 °C for 1 h on a clean bench. All ultrasonic cleaning was performed with a bath-type sonicator (Branson M2800-J, Emerson Electric, Missouri) operating at 110 W and an emission frequency of 40 kHz. Specimens prepared using a constant irradiation intensity of 170 mW cm^–2^ (measured under the glass plate covering the vat) and irradiation durations of 1, 3, and 10 min were denoted as T-1, T-3, and T-10, respectively. Specimens prepared at a constant irradiation time of 10 min and irradiation intensities of 30, 50, and 100 mW cm^–2^ were denoted as I-30, I-50, and I-100, respectively. All T- and I-series specimens were prepared by using a benzylation time of 3 h.

### Physicochemical Property Characterization

Fourier transform infrared (FT-IR) spectra were recorded by using a Nicolet iS20 spectrometer (Thermo Fisher Scientific, Waltham, MA) with an attenuated total reflectance (ATR) module. Freeze-fracture surfaces were coated with osmium and imaged by scanning electron microscopy (SEM; SU3500, Hitachi High-Tech, Tokyo, Japan) at 2 kV (for morphology observation) or 15 kV (for energy-dispersive X-ray spectroscopy (EDX) analysis). 3D confocal laser scanning microscopy (CLSM) measurements were performed by using a LEXT OLS5l00 instrument (Olympus, Tokyo, Japan). The average surface roughness (*S*_a_), interfacial expansion area ratio (*S*_dr_), and coating thickness (*d*) of the applied coating were calculated using 3D CLSM software.

The density of surface-coated pyridinium units was measured using the fluorescein staining method.^50^ The sample to be analyzed was immersed in 2 mL of 1% (w/v) fluorescein disodium salt solution (Tokyo Kasei Kogyo, Tokyo, Japan) for 10 min, washed with ultrapure water, sonicated in the same solution (5 mL) for 5 min, immersed in 2 mL of 0.1% (w/v) cetyltrimethylammonium chloride solution (Tokyo Kasei Kogyo, Tokyo, Japan), and sonicated for 20 min. Finally, the sample was treated with 100 mM phosphate buffer at pH 8.0 (10 vol %) (Fujifilm Wako Pure Chemical, Osaka, Japan), and the absorbance of the specimen at 501 nm was determined by UV/vis measurements (BioSpectrometer, Eppendorf, Hamburg, Germany) to calculate fluorescein concentration. The pyridinium unit density (mmol cm^–2^) was calculated as
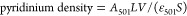
1where *A*_501_ is the absorption at 501 nm, ε_501_ is the extinction coefficient of fluorescein at 501 nm (77 mM^–1^ cm^–1^), *L* is the length of the polystyrene cuvette (1 cm), *V* is the volume of the extraction solution (2.2 mL), and *S* is the surface area of the specimen (1 cm^2^).

Water contact angles (WCAs; DM-501Hi, Kyowa Interface Science, Saitama, Japan) were measured using deionized water (drop size = 2 μL) at five different spots for each specimen, and the results were reported as the corresponding mean ± standard deviations (SDs). All WCAs were measured directly from photographs. Surface zeta potentials were determined by using an electrophoretic light-scattering spectrophotometer (ELSZ2, Otsuka Electronics, Osaka, Japan). Each specimen (1.5 cm × 3.7 cm) was set in a quartz cell and characterized according to a previously reported method^51^ using a dispersion of polystyrene latex particles (Otsuka Electronics, Osaka, Japan) with a mean diameter of 520 nm in 10 mM NaCl with pH 7.0 as a control. Three measurements were made for each specimen, and the results were reported as the corresponding means ± SDs.

### Viruses, Host Strains, and Media

Bacteriophages ϕ6 (NBRC 105899) and Qβ (NBRC 20012) were used as model enveloped and non-enveloped viruses, respectively, with *Pseudomonas syringae* (NBRC 14084) and *Escherichia coli* (NBRC 106373) as their respective hosts. The two bacteriophages were infected after incubation at 30 °C for *P. syringae* and 37 °C for *E. coli* until the logarithmic growth phase. Luria–Bertani (LB) medium (Formedium, Norfolk, UK) containing 2 mM calcium chloride (Ca-added LB medium) was used for the growth of bacteriophage-infected host cells, and culture plates were prepared by adding 1.5% (w/v) agar powder (Fujifilm Wako Pure Chemical, Osaka, Japan) to the Ca-containing LB medium. Additionally, 0.6% (w/v) agar powder was added to the Ca-added LB medium as the top agar for the plaque assay. The bacteriophage and host bacteria were obtained from the NITE Biological Resource Center (Chiba, Japan).

### Determination of the Viral Infection Titer

The viral infection titer was determined by the plaque assay using virus samples prepared by adjusting bacteriophage ϕ6 and bacteriophage Qβ dispersions to a concentration of 1.0 × 10^7^ plaque-forming units (PFU) with 1/500 nutrient broth medium (Becton Dickinson, Franklin Lakes, NJ). A 6 μL aliquot of the concentration-adjusted sample was placed on the specimen, covered with an 8 mm × 8 mm film, and allowed to react for a certain time. The contact reaction was stopped by adding the soybean-casein digest with lecithin and polysorbate 80 (SCDLP) medium (0.6 mL; Nippon Seiyaku, Osaka, Japan). The contact time was defined as the time from the addition of the virus solution to the addition of the SCDLP medium. Because polysorbate 80 in the SCDLP medium can inhibit the antimicrobial effect of quaternary ammonium salts,^52^ this medium was added to prevent further sample–virus interactions. Each solution was diluted with peptone-containing saline (Merck, Darmstadt, Germany), and a 10 μL aliquot of the diluted sample was added to 100 μL of the host culture medium in the log growth phase. After infection by the bacteriophage, upon 5 min of incubation at approximately 25 °C (bacteriophage ϕ6) or 37 °C (bacteriophage Qβ), top agar (4 mL) was added and layered on the bottom agar. The number of plaques that appeared after culturing was measured using a colony counter (Scan 500, Interscience, Saint-Nom-la-Bretèche, France).

### Polishing of PBVP-Coated PP Surfaces

The PBVP-coated PP samples were polished with a precision surface polisher (Handy Lap HLA-2, JEOL, Tokyo, Japan) using a load of 200 g and an alumina abrasive (grain size = 3 μm, 3M, Saint Paul, MN) wetted with distilled water. After polishing, the specimens were sequentially ultrasonicated in distilled water (30 s) and acetone (10 min) and vacuum-dried at 60 °C for 1 h. The total thickness of each specimen was measured before and after polishing using a digital micrometer (MDC-25MX, Mitutoyo, Kanagawa, Japan).

### Stability Test

The PBVP-coated PP (T-10) was exposed to heat, humidity, water, an organic solvent, and acid and alkali treatments. For the heat treatment, specimens were placed in a vacuum dryer (HD-15H, Ishii laboratory work, Osaka, Japan) at a constant temperature of 80 ± 2 °C for 60 and 133 h at rest. For the humidity treatment, specimens were placed in a small environmental test apparatus (SH241, ESPEC, Osaka, Japan) and held at 50 ± 2 °C and 95 ± 5% relative humidity for 60 and 133 h. For the aqueous organic solution treatment, specimens were immersed in 4 mL of water or 70% ethanol and allowed to stand at 25 °C for 15 h and 1 week (168 h). For the acid and alkali treatments, each specimen was immersed in 50 mL of 1 N HCl or 1 N NaOH and sonicated (Branson M2800-J) for 10 min. After treatment, each specimen was neutralized with the same concentration of NaOH and HCl and rinsed thoroughly with water. Each specimen was examined before and after each treatment by FT-IR (Nicolet iS20 spectrometer).

### Statistical Analyses

Dunnett’s tests were performed using statistical analysis software (KyPlot 6.0, KyensLab Inc., Tokyo, Japan) for cases in which significant differences were identified by the one-way analysis of variance. Differences at *p* < 0.05 were considered statistically significant. Data were presented as the means ± SDs.

## Results and Discussion

### Synthesis of PBVP-Coated PP

**Scheme 1:**
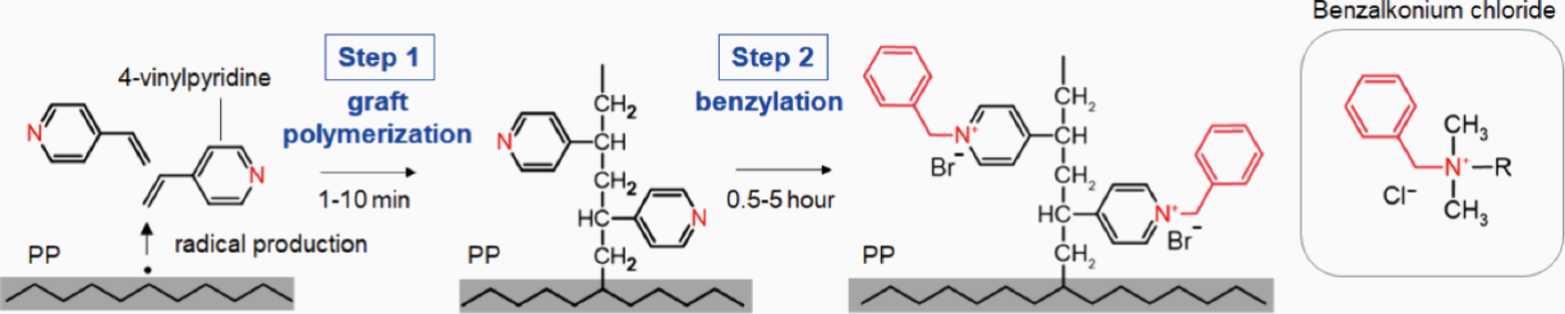
Schematic Synthesis of PP Surface-Grafted with PBVP As a result of UV excitation of benzophenone, radicals are generated by hydrogen withdrawal of PP, resulting in graft polymerization of 4-vinylpyridine. The benzylation of the grafted PVP with benzyl bromide produces a polymer with a benzalkonium chloride-like structure. The benzalkonium chloride skeleton is shown in red.

The binding of PBVP to the PP surface involved two steps: graft polymerization and benzylation (Scheme 1 and Figure S1). A mixture of benzophenone (sensitizer) and 4-vinylpyridine (monomer for grafting) was applied to the PP surface, and UV light-activated benzophenone abstracted hydrogen from the PP surface. Thus, the radicals reacted with 4-vinylpyridine to covalently bond to the surface (graft polymerization). Subsequently, the grafted PVP reacted with benzyl bromide to produce *N*-benzylammonium salts (benzylation). Given that the covalent bonding and polymerization of 4-vinylpyridine proceeded on the PP surface, the length of the polymer chain formed in the *z*-axis direction was expected to depend on the reaction time.

**Figure 2:**
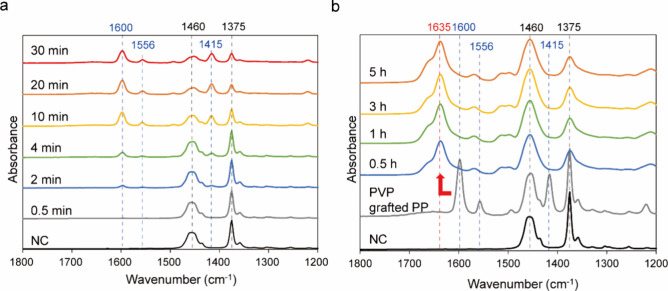
Fourier transform infrared (FT-IR) spectra of (a) poly(vinylpyridine) (PVP)-grafted PP prepared using different irradiation times and (b) PBVP-grafted PP prepared using an irradiation time of 10 min and different benzylation times. Peaks derived from PP, pyridine moieties, and pyridinium cations are shown as black, blue, and red dashed lines, respectively.

To optimize synthetic conditions, we examined the effect of UV irradiation time on the graft polymerization of 4-vinylpyridine using ATR-mode FT-IR spectroscopy. Figure 2a and Figure S2 show the spectra of poly(vinylpyridine) (PVP)-grafted PP specimens prepared by using various UV irradiation times. The bands at 2955 and 2871 cm^–1^ correspond to the stretching vibration of the methyl group of PP, and that at 2915 cm^–1^ can be attributed to the methylene group of PP. The peak at 1375 cm^–1^ indicates the C–H bending vibration on the carbon chain backbone of PP, whereas the peak at 1460 cm^–1^ indicates the scissor vibration of the methylene group of PP.^53^ The intensity of the pyridine ring (C=C and C–N) peaks at 1600, 1556, and 1415 cm^–1^^54^ increased with increasing UV irradiation time and plateaued when this time exceeded 20 min. These three peaks were ascribed to surface-bound PVP, as the specimens were sonicated in methanol after UV irradiation to remove any nonsurface-bound polymeric material. Correspondingly, the PP-derived absorption spectra (2955, 2915, 2871, 1460, and 1375 cm^–1^) showed a decrease in absorbance when the UV irradiation time and the absorbance remained almost the same after 10 min. Under the present treatment conditions, 4-vinylpyridine is considered to have been almost wholly consumed upon irradiation for more than 10 min.

Figure 2b and Figure S3 show the effects of the reaction time on the FT-IR spectra of *N*-benzylated samples and present the spectra of PVP-grafted PP before benzylation and pristine PP as controls. After benzylation, the characteristic 4-vinylpyridine peak shifted from 1600 to 1635 cm^–1^,^55^ which was attributed to the formation of 4-vinylpyridinium cations and confirmed the occurrence of benzylation. After 0.5 h of benzylation, the pyridine-derived peak at 1600 cm^–1^ disappeared and was replaced by the pyridinium peak at 1635 cm^–1^, which suggested that benzylation was complete after 0.5 h under our conditions. In this synthesis process, benzophenone was added as a sensitizer in the graft polymerization of 4-vinylpyridine. Comparing the FT-IR spectra of the coated and control samples confirms the presence of benzophenone residues in the coated samples as the 1660 cm^–1^ peak indicative of benzophenone was virtually absent in the spectrum of the grafted PP (Figure S4). However, since benzophenone was present in high concentrations during the grafting reaction, it may also contribute to the cross-linking reaction between the grafted chains of PBVP.

Based on these findings, we concluded that PP with covalently surface-bound PBVP (PBVP-coated PP) was successfully prepared by a combination of graft polymerization and benzylation. The synthesis of PBVP-coated PP was expected to be strongly influenced by the graft polymerization process because the covalent bonding of 4-vinylpyridine starts from the radicals formed on the PP surface.

### Physicochemical Properties of PBVP-Coated PP

**Figure 3:**
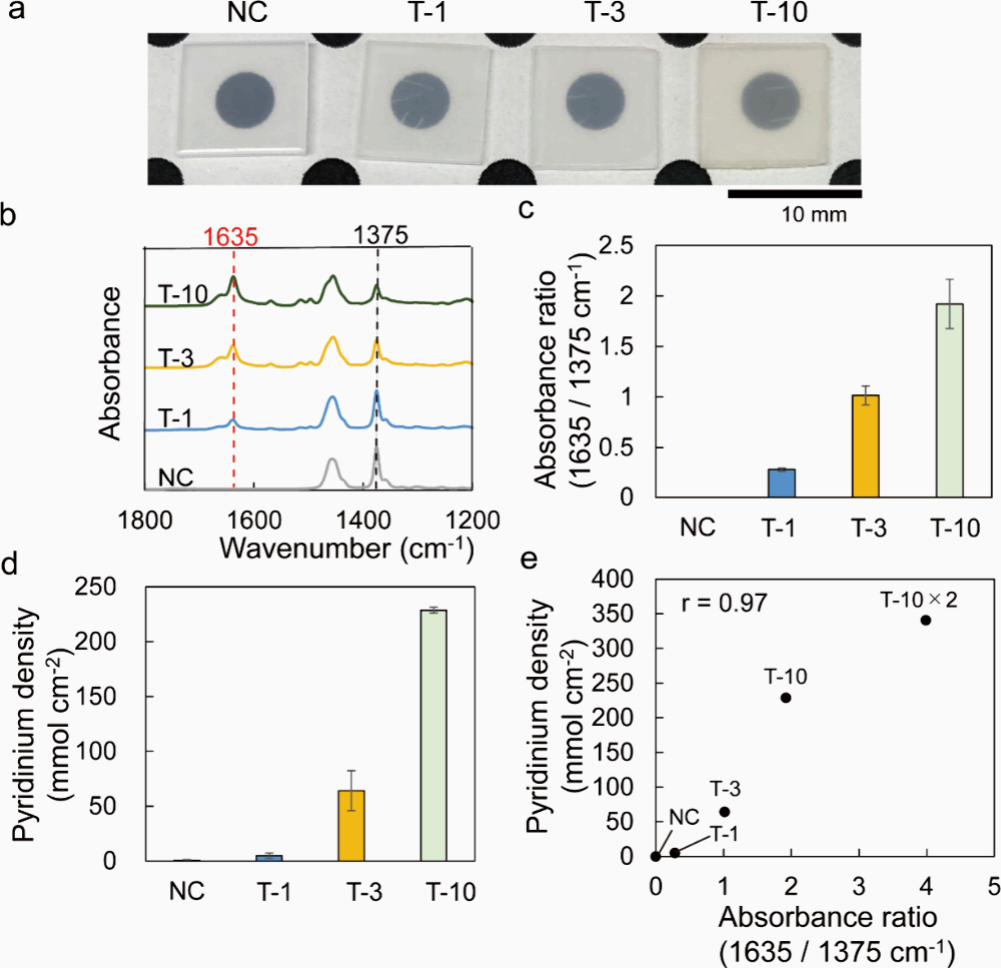
Characterization of compounds T-1, T-3, and T-10. (a) Photographs. (b) FT-IR spectra. Red and black dotted lines indicate pyridinium salt (1635 cm^–l^) and PP (1375 cm^–1^) peaks, respectively. (c) Absorbance ratios (means ± standard deviations (SDs) measured from 10 independent specimens). (d) Density of surface-coated pyridinium groups measured for three independent specimens. (e) Correlation between the absorbance ratio and the pyridinium density (*r* = 0.97). Data for T-10 × 2 are shown in Figure S5. NC denotes the noncoated control sample not subjected to graft polymerization and benzylation.

Figure 3 shows the properties of T-1, T-3, and T-10. These samples appeared slightly hazy compared to the noncoated control (NC). Although the haziness increased with increasing UV irradiation time, it was maintained at a level that allowed the underlying pattern to be recognized, and no significant discoloration was observed (Figure 3a). Figure 3b presents the FT-IR spectra of the specimens described above, revealing the presence of *N*-benzyl-4-vinylpyridinium moieties on the PP surface. For normalization, the absorbance ratio was calculated as
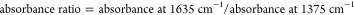
2

The absorbance ratio (Figure 3c) and the density of surface-bound pyridinium groups determined by the fluorescein staining method^50^ (Figure 3d) increased in the order of T-1 < T-3 < T-10. To further increase the pyridinium density, we repeated the 10 min grafting process and showed that the absorbance ratio and pyridinium density of the obtained sample (T-10 × 2) exceeded those of T-10 (Figure S5). Notably, the pyridinium density was linearly correlated with the absorbance ratio and could be estimated from the latter (Figure 3e). For the I-series specimens, the pyridinium density increased with increasing irradiation intensity (Figure S6).

**Figure 4:**
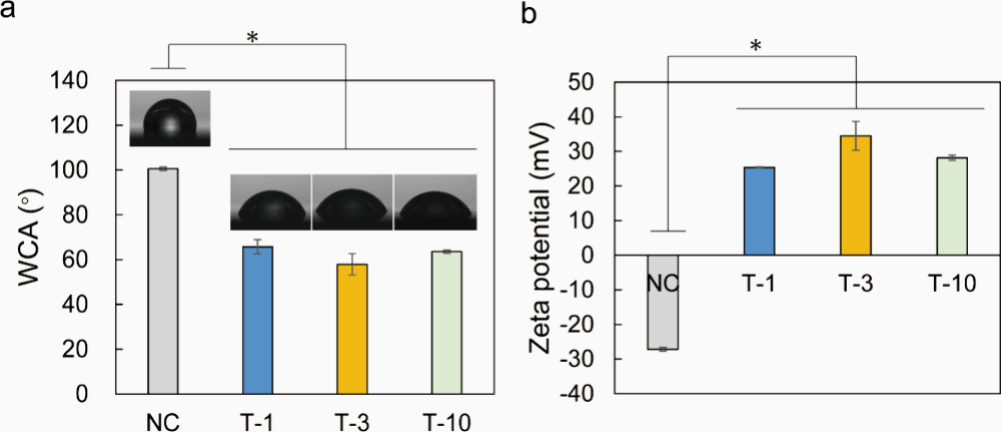
(a) Water contact angles (WCAs) of T-1, T-3, and T-10 and photographs of 2 μL water droplets thereon. Data represent the means and SDs for five different spots of the same sample. (b) Zeta potentials of the T-1, T-3, and T-10 surfaces. Data represent the means and SDs of three independent measurements performed for the same sample (*p < 0.05; Dunnett’s test). Statistical analysis compared NC to T-1, T-3, and T-10.

The WCA decreased (and hence surface hydrophilicity increased) in the order NC (100°), T-1 (65.7°), T-3 (57.9°), and T-10 (63.5°) (Figure 4a). Previous reports on methyl methacrylate bonded to PP surfaces by UV grafting revealed a similar WCA trend, indicating that the surface properties of PP change from hydrophobic to hydrophilic with increasing treatment time.^56^ The hydrophilization of the PP surface was ascribed to PBVP formation and indicated sufficient progress of graft polymerization. Although the pyridinium density was variable, this variation was not sufficiently large to affect the hydrophilic nature of the PP surface. The zeta potentials of PBVP-coated PP specimens were measured by an electrophoretic light scattering method based on the Laser-Doppler principle.^57^ The electro-osmotic flow profiles acquired with respect to the migration degree of polystyrene latex particles (Figure S7) were obtained according to the Mori and Okamoto formula^58^ and converted to zeta potentials using Smoluchowski’s formula^51^ (Figure 4b). T-1, T-3, and T-10 surfaces were found to be positively charged, whereas the NC surface was negatively charged, as described in a previous study^49^ on PP nonwoven fabrics. Moreover, the zeta potentials of T-1, T-3, and T-10 were close to the value previously reported for a quaternary poly(4-vinylpyridine)-fixed natural latex film.^59^ Given that virus surfaces are negatively charged under neutral conditions^60^ because of the nature of their constituent proteins and phospholipids, the positive surface charge of the PBVP coating may facilitate virus–interface interactions.

### Analysis of PBVP-Coated PP Surfaces and Coating Layers

**Figure 5:**
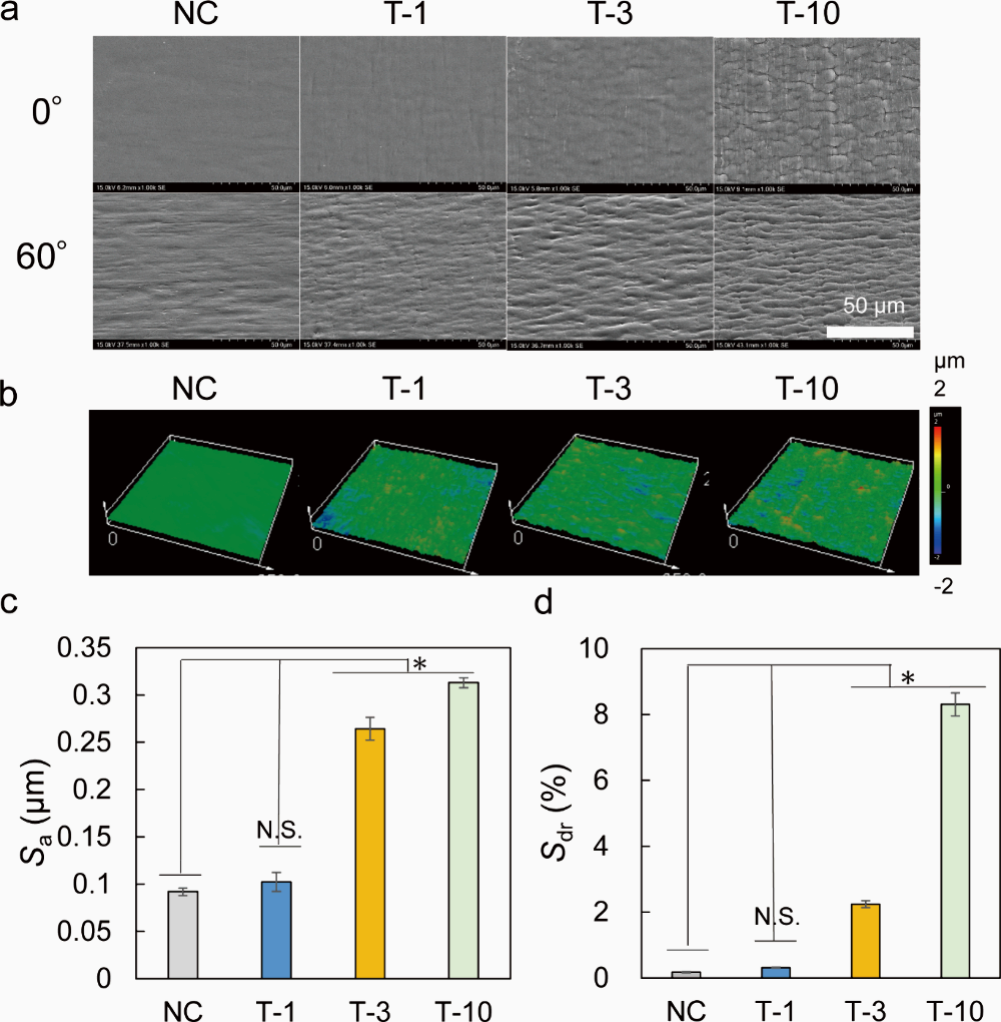
(a) Scanning electron microscopy (SEM) images of T-1, T-3, and T-10. Top and bottom rows show top views at 0° and bottom views at 60°, respectively. (b) Surface images of T-1, T-3, and T-10 acquired using three-dimensional confocal laser scanning microscopy (3D CLSM; 50× objective, ∼250 μm × 250 μm). (c) Average surface roughness (*S*_a_) and (d) interfacial expansion area ratios (*S*_dr_) of T-1, T-3, and T-10 calculated from 3D CLSM results. All data are represented as the means ± SDs (*p < 0.05; Dunnett’s test). N.S. = not significant.

The surface morphology of the PBVP-coated PP was observed by SEM (Figure 5a) and 3D CLSM (Figure 5b). Figure 5a shows that NC had a smooth surface, whereas grain boundaries were observed in PBVP-coated PP, confirming that the grafted resin densely covered the PP surface. As expected, surface roughness increased with increasing UV irradiation time for continuous graft polymerization on the PP surface. The results of 3D CLSM resembled those of SEM. No significant difference between NC and T-1 was observed regarding *S*_a_ (Figure 5c). However, with increasing UV irradiation time, an increase in unevenness height was observed for T-3 and T-10, and the corresponding *S*_dr_ values were ∼8% higher than that of NC (Figure 5d). This finding suggests that benzylation occurred in the *z*-axis direction on the PP surface, and it is related to the fact that the increase in the pyridinium density shown in Figure 3c could not be explained by benzylation of the surface alone.

**Figure 6:**
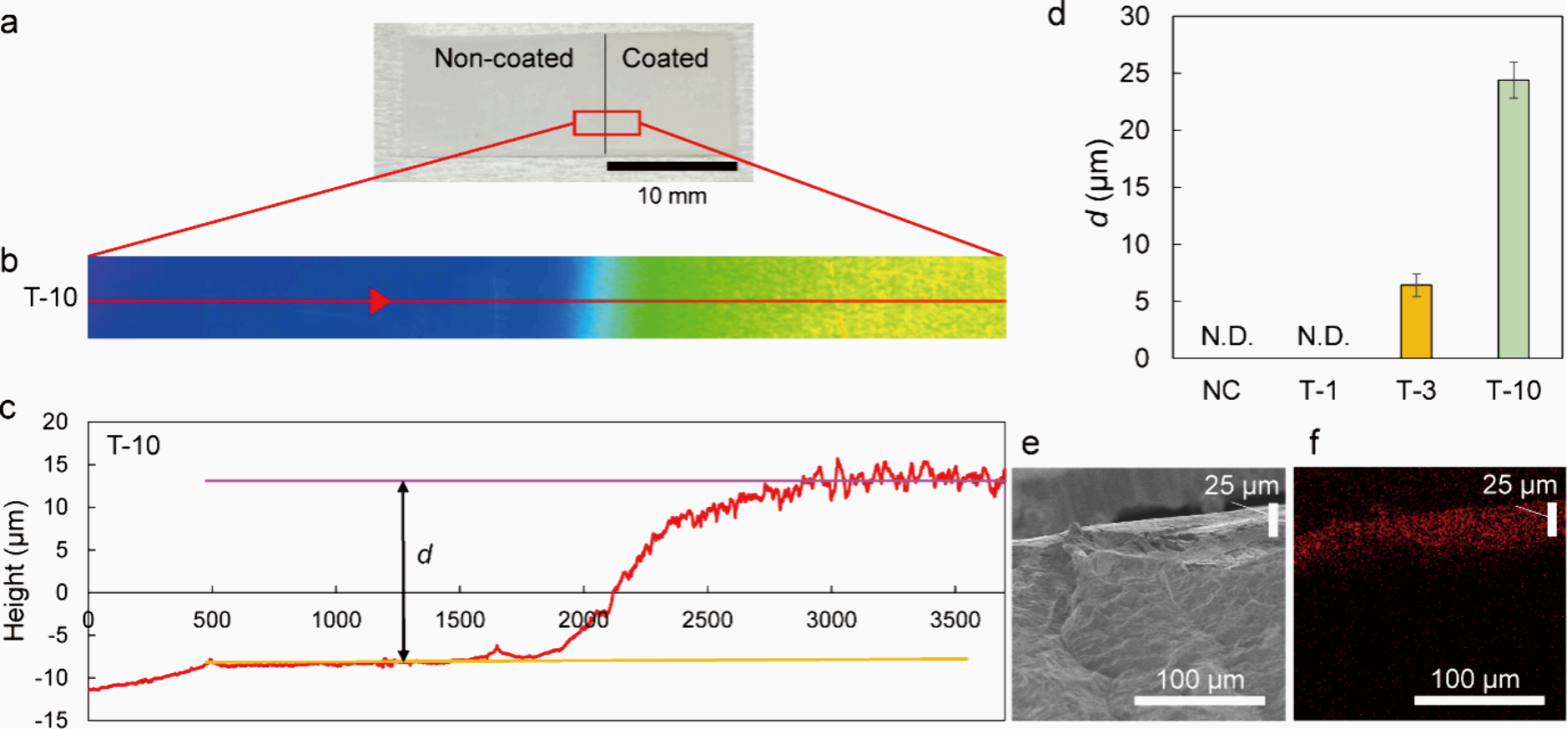
(a) PBVP-coated PP specimen divided by a black vertical line into non-coated (left) and coated (right) parts. The observation area (red frame) was centered on the border to ensure that both areas were the same. The scale bar equals 10 mm. (b) Two-dimensional image of the red-frame area from panel (a), with color indicating height information. The red line indicates the line scan position. (c) Surface topography of the area line-scanned in panel (b). Horizontal and vertical axes display the *x*-coordinate and height, respectively. The horizontal orange and purple lines indicate the noncoated area and unevenness in the coated area, respectively. The difference in height between these two lines was used to calculate *d*. (d) Values of *d* measured for T-1, T-3, and T-10 by 3D CLSM. Data represent the means and SDs of measurements performed at three different locations of the same sample. N.D. = not detected. (e) Cross-sectional SEM image of T-10. (f) Distribution of bromine (red) for the SEM image in panel (e).

To investigate the correlation between *d* (Figure S8) and the surface roughness of T-1, T-3, and T-10, coated and noncoated areas were created on the same specimen, and step analysis was performed using 3D CLSM. Specimens for this analysis were prepared by carrying out graft polymerization and benzylation reactions on a PP substrate with the left half covered by masking tape and aluminum foil, followed by thorough washing after the end of the process series (Figure 6a). To create a comprehensive overview, we compiled 16 images by selecting a specific observation area (approximately 250 μm × 250 μm). Eight images were consecutively captured on each side centered on the boundary between both areas. The two-dimensional images of PBVP-coated PP samples and the surface topographies of the line-scanned areas were used to determine *d* (Figure 6b,c and Figure S9). Specifically, *d* was calculated from the step difference between both areas and was approximately 6.4 and 24.4 μm for T-3 and T-10, respectively (Figure 6d). Although *d* could not be measured for T-1, it was inferred to be below the detection threshold because previous analyses (Figures 3 and 4) suggested the presence of PBVP on the surface of this sample. The increase in *d* with increasing UV irradiation time was consistent with the concomitant increase in pyridinium density, indicating that PBVP was synthesized in the *z*-axis direction on the PP surface.

SEM-EDX of the cross section was conducted to examine the distribution of pyridinium within the coating layer. This technique allows for elemental mapping while observing the morphology and offers superior spatial resolution compared with X-ray photoelectron spectroscopy. T-10 was frozen and then broken; the obtained surface was analyzed by SEM-EDX. During the synthesis of PBVP-coated PP, 4-vinylpyridine is benzylated with benzyl bromide. As bromine is expected to be present in the coating as a counterion to the pyridinium group, we examined the distribution of bromine by SEM-EDX. The EDX mapping of an arbitrarily selected area in the SEM image shown in Figure 6e revealed the presence of bromine in the area identified as the PBVP coating but not in the area identified as PP (Figure 6f and Figure S10). The uniform distribution of the counterion bromine was observed in the PBVP coating layer.

### Chemical Stability of the Coating Layers

**Figure 7:**
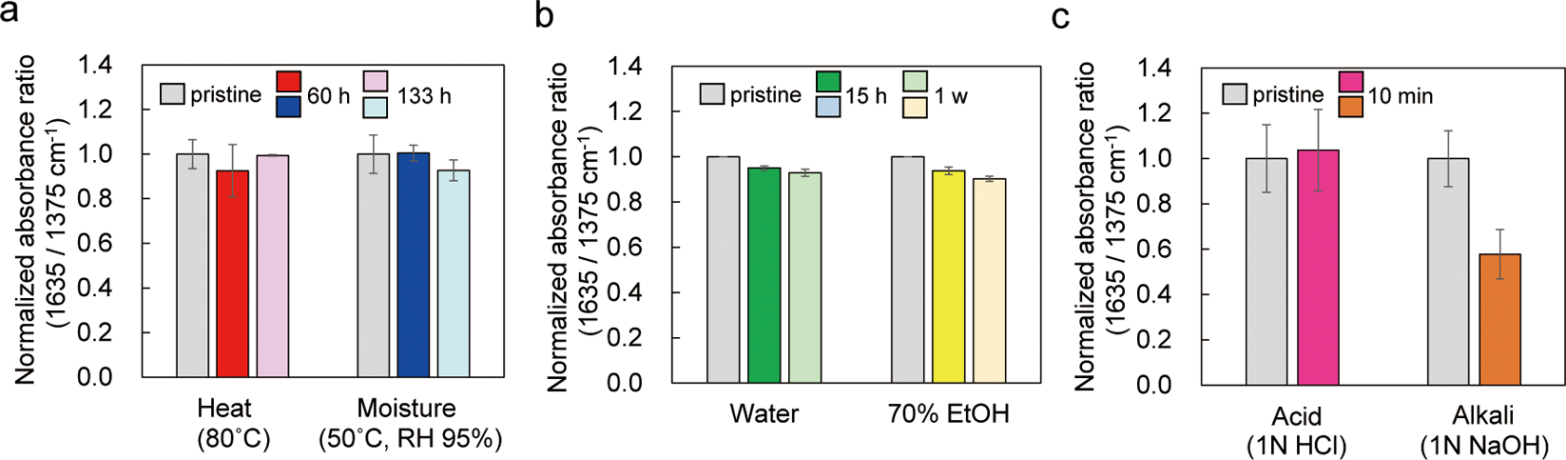
Chemical stability evaluation with normalized absorbance ratio (1635/1375 cm^–1^) by FT-IR. The normalized value for the pristine samples is set to 1. (a) Heat and moisture treatments, (b) water and 70% ethanol immersion, and (c) acid and alkali treatments. All data are represented as the means ± SDs of three independent measurements.

An important consideration in the handling of surface-coated materials is the leaching of compounds from the surface over time, which requires an understanding of chemical stability and human safety under a variety of environmental conditions. The effects of heat, humidity, water, organic solvent, acid, and alkali treatments on the surface-grafted films of PBVP were evaluated based on changes in the FT-IR spectra before and after these treatments. Figure 7 and Figure S11 show the relative changes in the absorbance ratios of pyridinium salts (1635 cm^–1^) and PP (1375 cm^–1^) for T-10 specimens treated under six different conditions. Heat treatment at 80 °C and 95% humidity for 133 h led to only a slight decrease in the absorbance ratio of the pyridinium groups bound to the PP surface, and the water and 70% ethanol treatments also showed a similar trend at 1 week. Thus, at least in the environments tested in this study, the amount of pyridinium groups did not significantly decrease and remained relatively stable. These results were expected because the grafted polymer was tightly bound to the PP surface by covalent bonds. However, the 1 N NaOH treatment reduced the absorbance ratio by approximately 40%. After this alkaline treatment, the baseline peak at 1600 cm^–1^ in the FT-IR spectrum increased; the Br^–^ and benzyl groups comprising the grafted films are assumed to have reacted readily with Na^+^ and OH^–^ (Figure S12). Since the coronavirus pandemic, the consumption of QACs has skyrocketed worldwide. QACs are relatively safe antibacterial and antiviral agents that are added to a variety of hygiene products, but there are also concerns about how large quantities of disposed QACs may adversely affect the human body and the environment.^8,61^ These chemical stability tests are only a limited assessment of the coatings, however, and a more multifaceted assessment will be required, given these safety concerns.

### Antiviral Effect of PBVP-Coated PP

**Figure 8:**
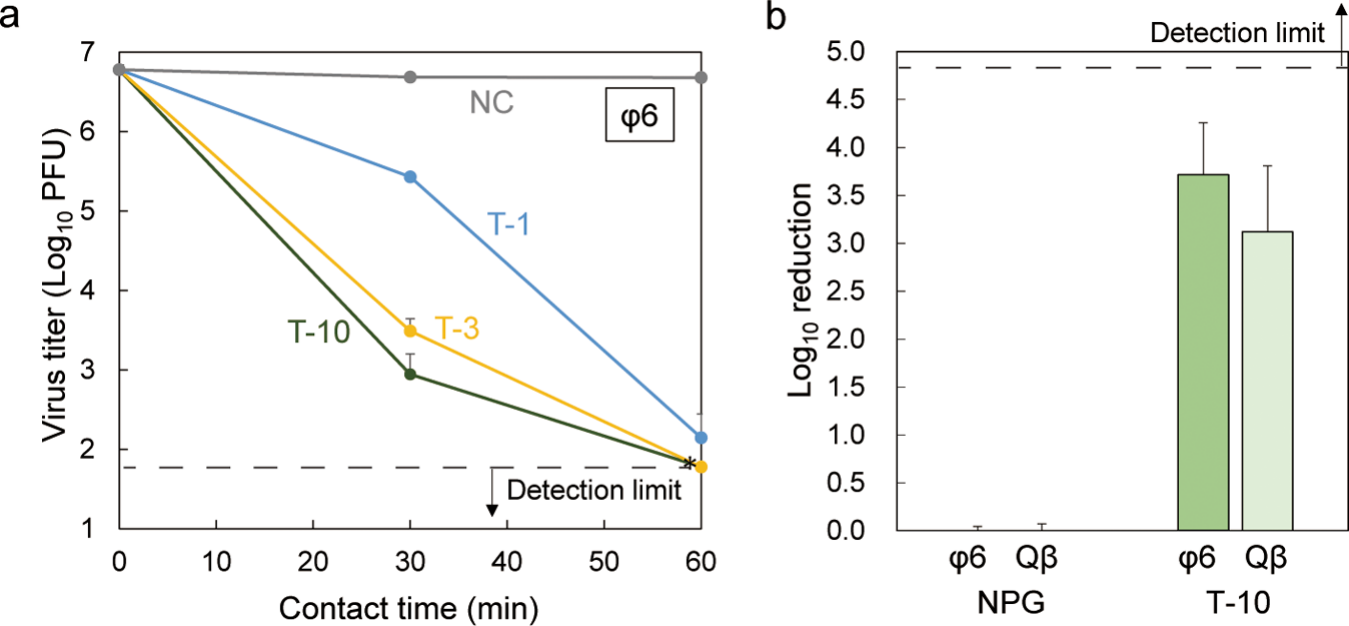
(a) Antiviral effects of T-1, T-3, and T-10. Time course of the viral titer for bacteriophage ϕ6. (b) Comparison of antiviral activity after 30 min of contact with bacteriophages ϕ6 and Qβ. Log_10_ reduction indicates the logarithmic reduction in the virus titer after 30 min of contact between T-10 and NC and each bacteriophage. As another control sample, PP treated by the same process as that for T-10 but without the addition of 4-vinylpyridine (hereafter denoted by NPG) was also examined. Data are represented as the means ± SDs of three independent measurements. The asterisk (*) and dotted black line indicate virus titers under the detection limit.

Materials with polycationic coatings are known to disrupt the membrane of the envelope virus,^62^ releasing its internal nucleic acids and rendering it less infectious to the host. Hence, PBVP-coated PP was expected to have antiviral properties, which were evaluated by a plaque assay using bacteriophage ϕ6 as an enveloped virus model. A dispersion of this virus, adjusted to a viral infection titer of ∼1.0 × 10^7^ PFU, was dropped onto the specimen and allowed to maintain contact with the same for 30–60 min at approximately 25 °C. The recovered virus solution was infected with the host bacteria, *P. syringae*, and the infection titer was determined from the PFU (Figure S13). The antiviral effects of T-1, T-3, and T-10 are shown in Figure 8a. In all cases, the infectivity titer of the virus decreased with increasing contact time. The strongest antiviral effect was observed for T-10, in which case the infectivity titer decreased by more than 3 orders of magnitude after 30 min, whereas contact for 60 min resulted in a drop to values below the detection limit (60 PFU), decreased by approximately 5 orders of magnitude of the plaque assay. A previous study reported approximately 3 orders of magnitude after 60 min of contact between a PP nonwoven fabric-grafted QA polymer and an envelope virus (murine hepatitis virus-A59).^49^ The virus types and assay methods are different, making a comparison of antiviral activity difficult. A decrease to below the detection limit after 60 min of contact was also observed for T-3, which was slightly less active than T-10. Both specimens showed a more than 2.5-fold difference in pyridinium density, and *S*_a_, *S*_dr_, and *d* were higher for T-10. However, the difference in the antiviral effects between the two specimens was minimal. Possibly, the number of surface PBVP molecules of T-10 capable of contacting the virus had already reached its maximum, while the virus could not contact the PBVP located deeper in the coating. Conversely, T-1 showed a reduction in viral infection titer of more than 4 orders of magnitude after 60 min of contact but featured lower reaction rate than T-3 or T-10. The only difference between the three types of specimens was the UV irradiation time used for graft polymerization. The substantial effect of this small difference on the antiviral effect is significant and can be used to control the antiviral performance of PP surfaces.

To investigate the effect of nucleic acid levels on the antiviral action of PBVP-coated PP, we adopted a reverse transcription–quantitative polymerase chain reaction method using virus solutions in contact with the material surface. Oligonucleotide primers were designed to target the S_1 genes of bacteriophage ϕ6, and the number of RNA copies of the virus in contact with the PBVP-coated PP was quantified. In the case of T-10, the number of RNA copies decreased with an increase in contact time (Figure S14). As QACs contribute to the degradation of viral surface proteins and RNA,^63^ a similar mechanism was expected for the PBVP-coated PP.

For comparison with that of bacteriophage ϕ6, the antiviral activity of bacteriophage Qβ, a model non-enveloped virus, was also evaluated. Specifically, the log_10_ reduction, which represents the logarithmic reduction in viral titer to bacteriophage Qβ, was determined after 30 min of contact with the T-10 specimen under the same test conditions. As shown in Figure 8b, the log_10_ reduction of bacteriophage ϕ6 was greater than that of bacteriophage Qβ for T-10. In general, QACs inactivate enveloped viruses, but are less likely to inactivate non-enveloped viruses. QAC-coated surfaces interact with the lipid bilayer of enveloped viruses, causing envelope destruction and viral inactivation. By contrast, non-enveloped viruses do not have a lipid bilayer and interact only with hydrophilic protein capsids, which do not completely destroy the viral particles and are therefore considered to have low antiviral activity.^64^ Since the efficacy of benzalkonium chloride, one of the QACs, against non-enveloped viruses has been reported to vary with concentration and virus species,^65^ the present findings are only a phenomenon limited to bacteriophage Qβ.

### Correlation Between *d* and Antiviral Effect

**Figure 9:**
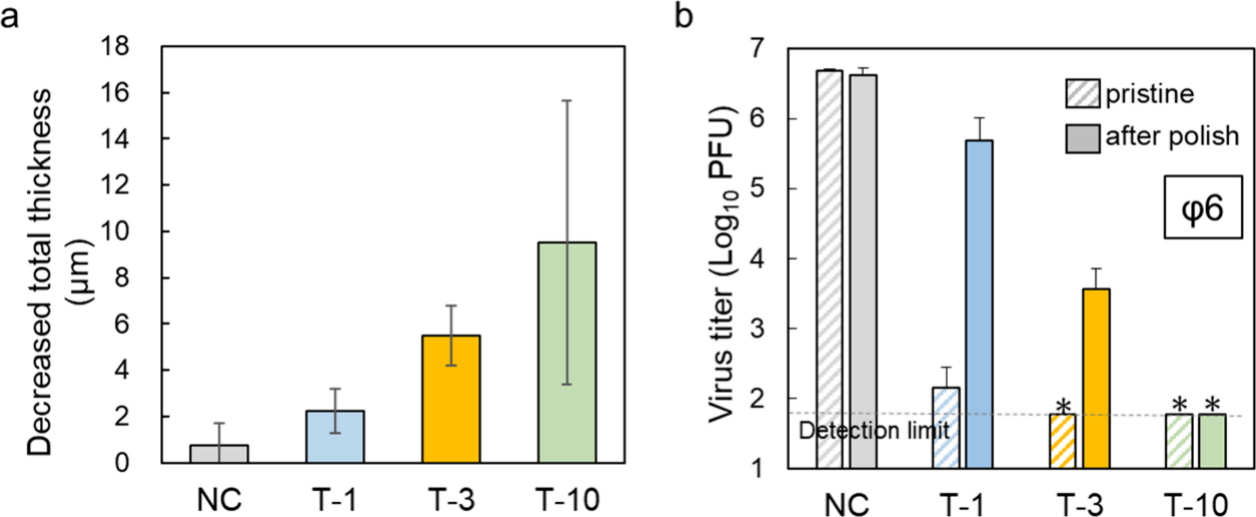
(a) Decrease in the total thickness of polished T-1, T-3, and T-10 measured by a digital micrometer. (b) Antiviral effects of polished T-1, T-3, and T-10 after 60 min of contact with bacteriophage ϕ6. All data are represented as the means ± SDs of three independent measurements. Asterisks (*) and dotted black lines indicate virus titers under the detection limit.

PBVP-coated PP was expected to maintain its antiviral effect even when scratched or abraded, as the QACs were hypothesized to be distributed on the PP surface in a 3D manner (Figure 6f). To prove this hypothesis, we polished T-1, T-3, and T-10 using a precision surface polisher until the surface was smooth and examined the antiviral effect of the polished specimens. The FT-IR spectra of all polished samples (Figure S15) featured a pyridinium salt-derived peak at 1635 cm^–1^, indicating a decrease in residual PBVP. The safety of this polishing product must be confirmed in the future. Figure 9a shows the decrease in the total thickness after polishing. Four NC PP specimens were examined with a digital micrometer, and the mean thickness was used as the substrate thickness. Subsequently, *d* was estimated by subtracting substrate thickness from total thickness (Figure S16a) and used to calculate residual coating thickness as
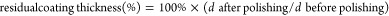
3

The residual coating thickness values were calculated as ∼90% for T-1 and ∼80% for T-3 and T-10 (Figure S16b). The surface condition of each specimen after polishing was reconfirmed by 3D CLSM. As shown in Figure 5, the surface roughness increased with increasing reaction time. However, *S*_a_ and *S*_dr_ decreased after polishing, in line with the concomitant reduction in surface roughness (Figure S17). Among the specimens polished using 40 round trips with a load of 200 g, T-10, which had the thickest PBVP coating, was the most affected, showing a higher reduction in *d*.

The antiviral effect of polished specimens was evaluated by a plaque assay. A dispersion of bacteriophage ϕ6 adjusted to a viral infection titer of ∼1.0 × 10^7^ PFU was dropped onto each specimen, and the solution was used to infect *P. syringae* after 60 min of contact. The infection rate was calculated by counting the plaques (Figure 9b). The NC PP did not show any change in the viral infection titer before and after polishing, which confirmed that polishing does not affect the antiviral effect. In the case of T-1, which had the lowest *d*, polishing significantly reduced the antiviral effect, possibly by decreasing the amount of PBVP bound to the surface. However, compared to NC, polished T-1 showed an antiviral effect higher by about 1 order of magnitude. The FT-IR spectrum of polished T-1 showed an absorption peak originating from pyridinium salts (Figure S15), thus suggesting that the antiviral effect was retained, albeit weakly. The antiviral effect of T-3 was also reduced by polishing. However, the reduction was less pronounced than in the case of T-1, and the infectivity titer decreased by approximately 3 orders of magnitude after 60 min of contact, which indicated that the antiviral effect was retained. Thus, polished T-1 and T-3 could further reduce the viral infection titer at longer contact times. T-10 behaved differently from T-1 and T-3, retaining the ability to reduce virus infection titers to below the detection limit (60 PFU) after 60 min of contact, even after polishing. The PBVP coating of T-10 was the thickest among the three samples and retained approximately 80% of its thickness even after polishing, which may explain this behavior. Moreover, the retention of antiviral capacity was proportional to the coating thickness; even the inner layer of the PBVP coating showed the same level of antiviral effect. This finding is consistent with the results of cross-sectional SEM-EDX analysis, which showed that bromine (counteranion of the pyridinium group) was uniformly distributed in the coating layer (Figure 6f). Therefore, the PBVP-coated PP was concluded to be a material consistent with our concept (Figure 1), as PBVP containing QACs was synthesized in a 3D form following the benzylation reaction and QACs were abundantly present within the coating layer. However, the correlation between surface roughness, surface area of PP, and antiviral effect is still unclear and should be elucidated in future works, which should also focus on the physicochemical interactions between the virus and the polymeric material surface. The increased WCAs of T-1, T-3, and T-10 after polishing (Figure S18) indicated a decrease in the surface energy, which may have made it more difficult for viruses to adhere to the surface, thereby reducing antiviral activity. Previous studies have shown a similar relationship between antiviral properties and the material surface roughness and wettability.^66^

## Conclusions

PP is used in many commonly encountered materials, such as automotive parts, textiles, electrical products, medical equipment, and packaging materials. The recent COVID-19 pandemic has triggered a growing need for antiviral materials; however, imparting antiviral properties to PP has remained technically challenging because of its low polarity and surface energy. Herein, we realized antiviral PP with a 3D surface coating of PBVP containing QACs. The simple synthesis of this material (several minutes of UV irradiation and a benzylation reaction of 0.5–5 h) can be easily scaled up if the appropriate facilities are available. Furthermore, our approach potentially applies to other olefinic polymers such as polyethylene and polybutene. Moreover, previous pioneering studies showed that PBVP has high antimicrobial efficacy against Gram-negative and Gram-positive bacteria; thus, PBVP-coated PP is also expected to exhibit antimicrobial activity. The most significant feature of PBVP-coated PP is its ability to maintain stable antiviral performance despite scratches and abrasion at a sufficiently large *d*, which did not exceed ∼25 μm in the present study. Long-term stability, particularly resistance to scratching and abrasion, is essential for practical applications of antiviral materials, highlighting the value of our approach. Additionally, QACs introduced onto polymer surfaces by covalent bonding may allow the consumption of organic solvents to be reduced, unlike that with conventional ethanol or surfactant sprays. However, *d* is likely limited, and prolonged graft polymerization will lead to transparency loss. Antiviral properties are related not only to *d* and pyridinium density but also to the shape and physicochemical properties of the PP surface. Thus, further optimization of antiviral polymers, including PBVP, is needed to prevent the next pandemic.

## Supporting Information Available

Additional FT-IR spectra, microscopic images, thickness profiles, RT-qPCR data, electro-osmosis profiles, and other sample characterizations (PDF)

## Abbreviations

ATR: attenuated total reflectance
CLSM: confocal laser scanning microscopy
COVID-19: coronavirus disease 2019
EDX: energy-dispersive X-ray spectroscopy
FT-IR: Fourier transform infrared
LB: Luria–Bertani
NC: non-coated control
PFU: plaque-forming unit
PP: polypropylene
PBVP: poly(*N*-benzyl-4-vinylpyridinium bromide)
PVP: poly(vinylpyridine)
QAC: quaternary ammonium compound
SCDLP: soybean-casein digest with lecithin and polysorbate 80
SD: standard deviation
SEM: scanning electron microscopy
UV: ultraviolet
WCA: water contact angle
3D: three-dimensional
