# Supplementary material for: Polypropylene-Rendered Antiviral by Three-Dimensionally Surface-Grafted Poly(N-benzyl-4-vinylpyridinium bromide)
Source: ACS Appl Mater Interfaces. 2024 Feb 12;16(8):10590–600. doi: 10.1021/acsami.3c15125 (PMC10910468; doi:10.1021/acsami.3c15125)
Supplement: Supplementary file 1 — am3c15125_si_001.pdf [file am3c15125_si_001.pdf]

Supporting Information

Polypropylene Rendered Antiviral by Three-Dimensionally Surface-Grafted Poly(*N*-benzyl-4-vinylpyridinium bromide)

Rie Hirao, † Hisato Takeuchi, † Jumpei Kawada, Nobuhiro Ishida*

Toyota Central R&D Labs, Inc., Nagakute, Aichi, 480-1192 Japan

* E-mail: n-ishida@mosk.tytlabs.co.jp

† These two authors contributed equally to this work.

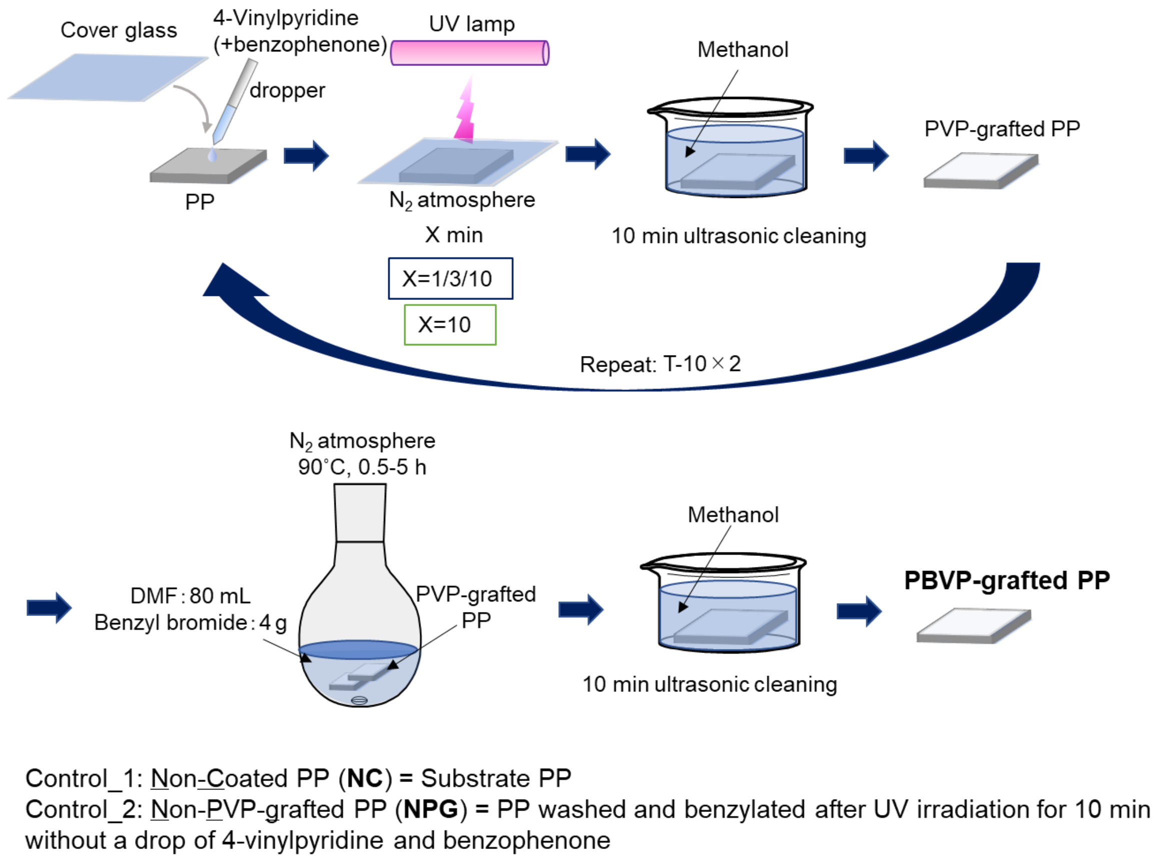

Figure S1. PBVP coating formation on PP. PVP = poly(4-vinylpyridine), DMF = dimethylformamide, PBVP = poly (*N*-benzyl-4-vinylpyridinium bromide). Non-coated substrate PP was designated as NC, and the PP that was washed and benzylated after UV irradiation for 10 min without any 4-vinylpyridine or benzophenone was designated as NPG.

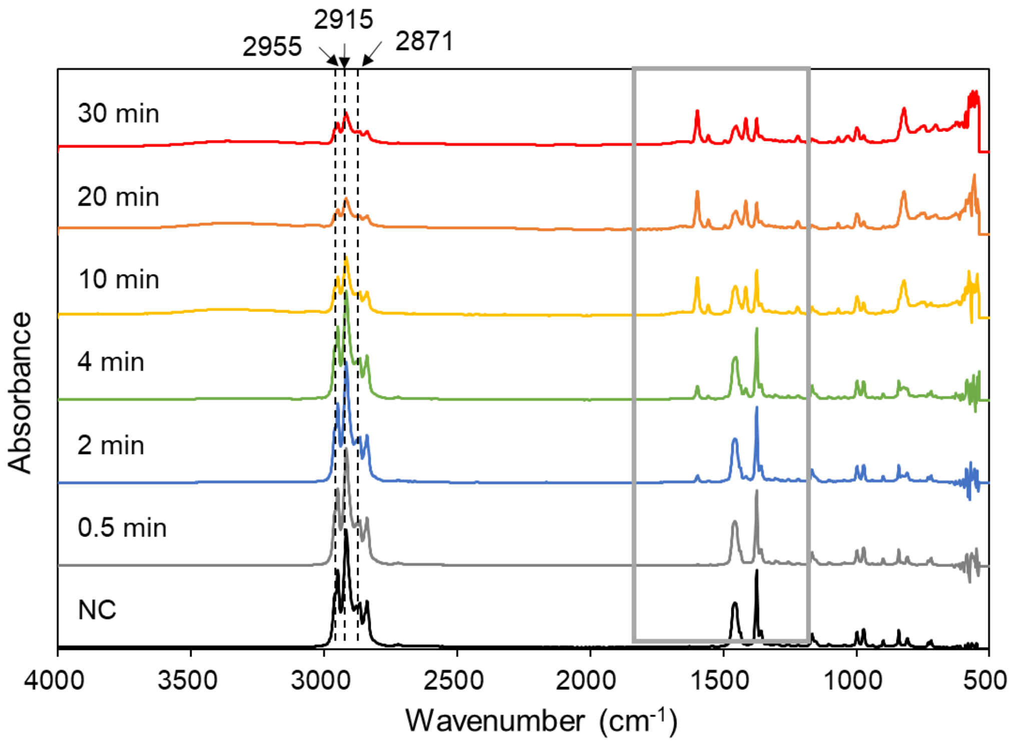

Figure S2. FT-IR spectra of PVP-grafted PP prepared by varying the UV irradiation time from 0.5 to 30 min. Peaks derived from PP (2955, 2915, and 2871 cm^{-1})[1] are shown as black dashed lines. The inner portion of the gray frame is enlarged and shown in Figure 2a.

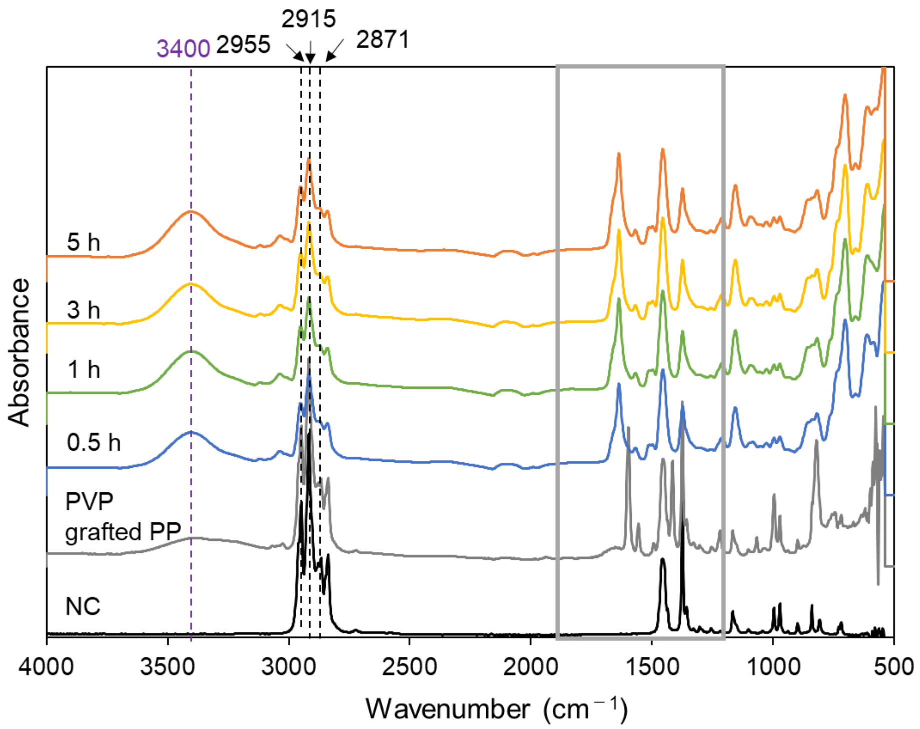

Figure S3. FT-IR spectra of PBVP-grafted PP prepared by varying benzylation times from 0.5 to 5 h. The absorption peak at 3400 cm^{-1} (purple dashed line) is caused by adsorbed water present in both PVP- and PBVP-grafted PP. Peaks derived from PP (2955, 2915, and 2871 cm^{-1}) are shown as black dashed lines. The inner portion of the gray frame is enlarged and shown in Figure 2b.

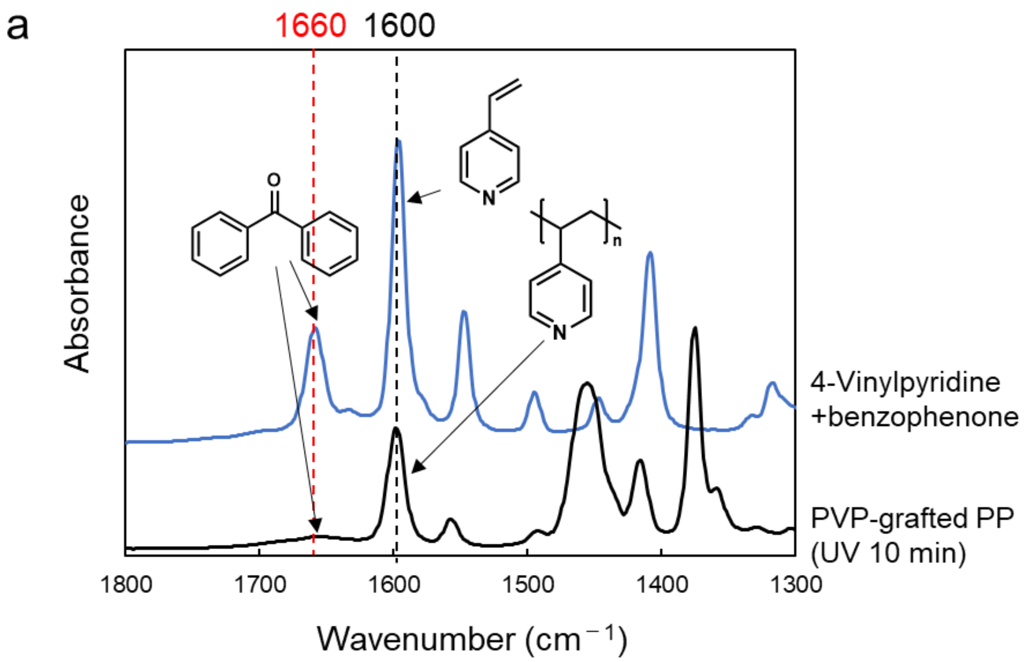

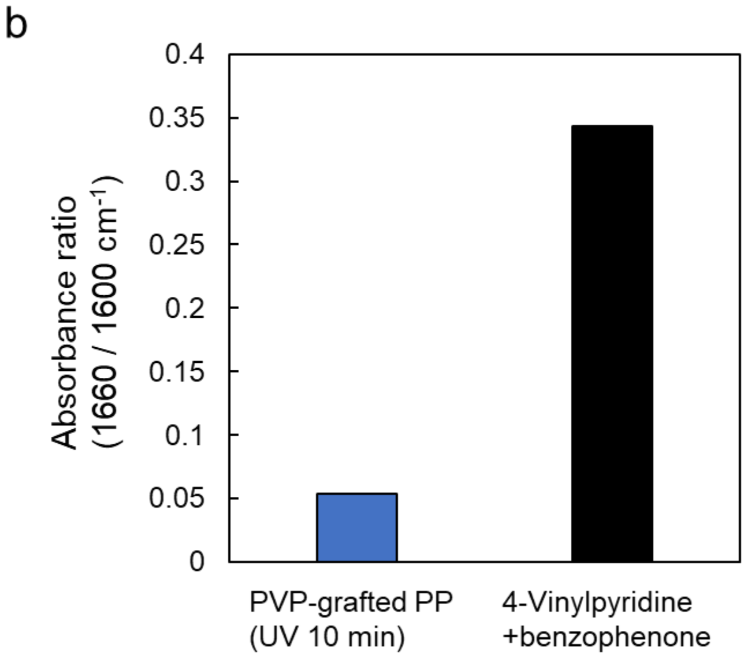

Figure S4. (a) FT-IR spectra of a mixture of 4-vinylpyridine and benzophenone and PVP-grafted PP (UV irradiation 10 min). Red and black dotted lines indicate benzophenone (1660 cm^{-1}) and pyridine (1600 cm^{-1}) peaks, respectively. (b) Absorbance ratio between the benzophenone (1660 cm^{-1}) and pyridine (1600 cm^{-1}) peaks for each specimen.

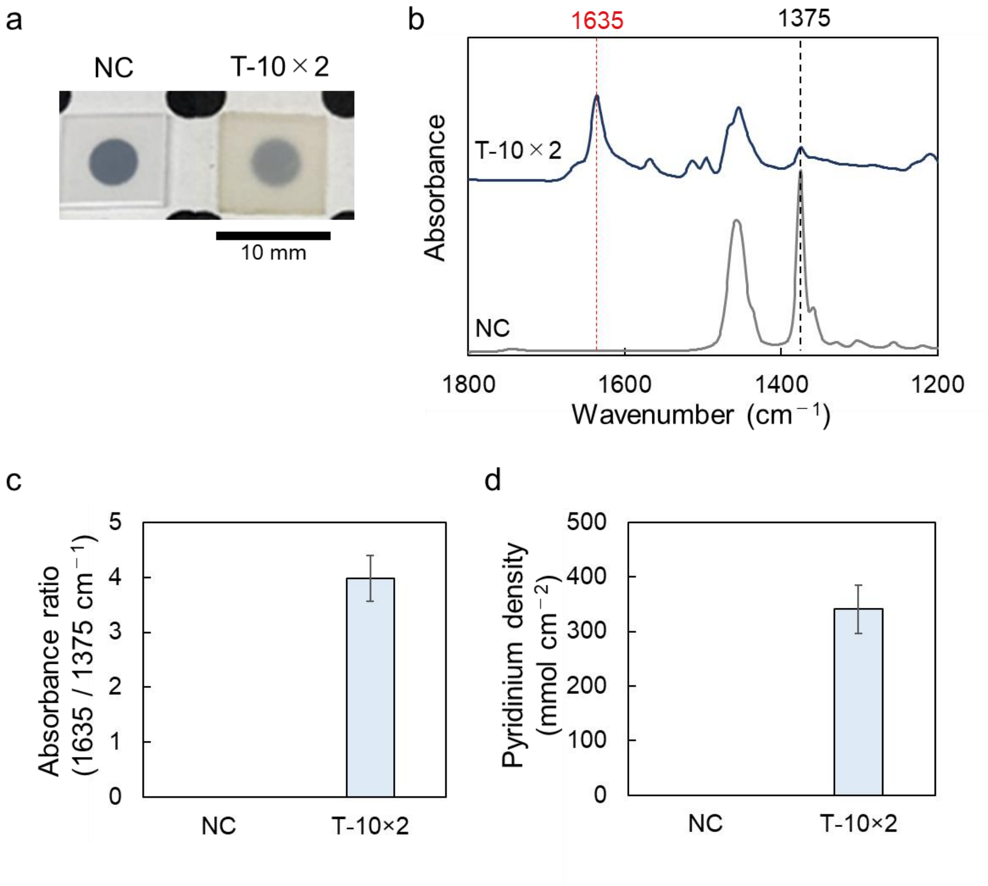

Figure S5. (a) Photographs of T-10×2. NC indicates control without graft polymerization and benzylation treatment. Transparency was maintained for all specimens. (b) FT-IR spectrum of T-10×2. Red and black dotted lines indicate pyridinium salt (1635 cm^{-1}) and PP (1375 cm^{-1}) peaks, respectively. (c) Ratio of pyridinium salt (1635 cm^{-1}) to PP (1375 cm^{-1}) absorbances for each specimen. Data are presented as the means ± SDs measured from 10 different specimens. (d) Surface-coated pyridinium group density (mmol cm^{-2}) measured by the fluorescein staining method.[2] Three independent specimens were examined.

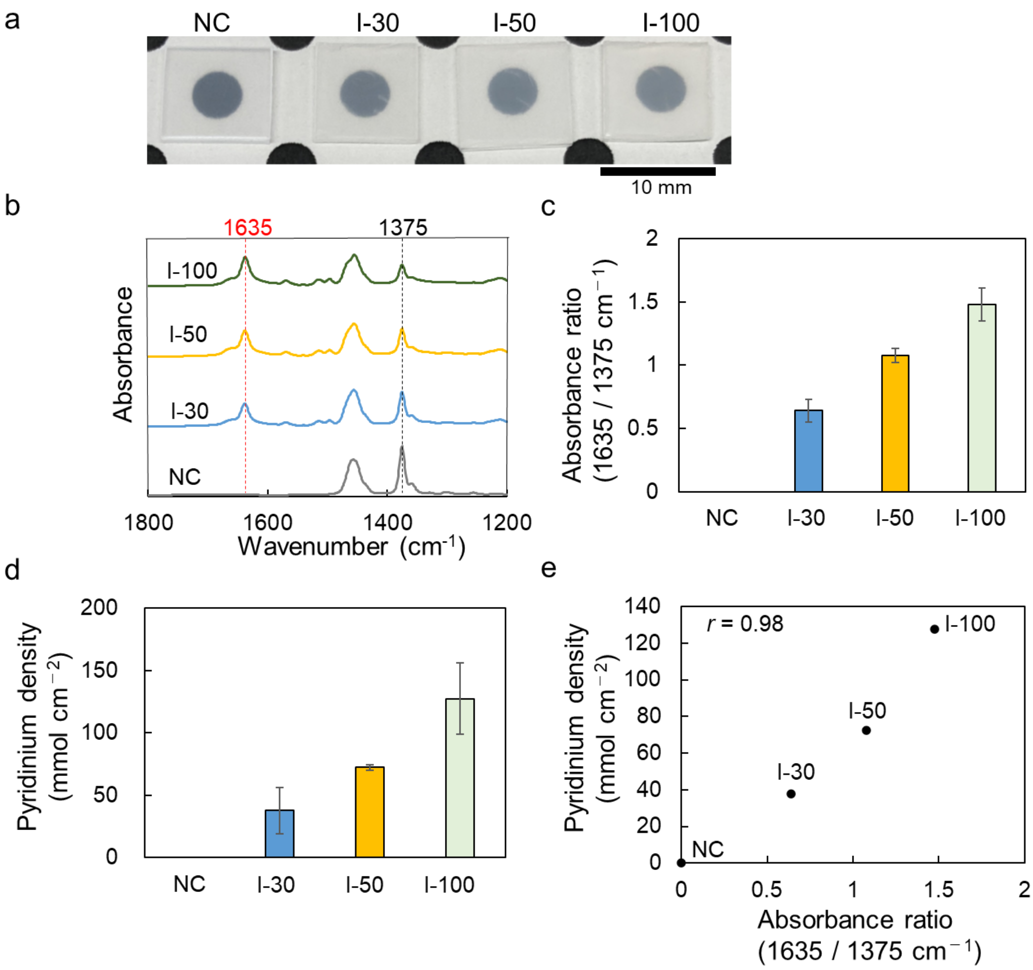

Figure S6. (a) Photographs of I-30, I-50, and I-100. NC indicates control without graft polymerization and benzylation treatment. Transparency was maintained for all specimens. (b) FT-IR spectra of I-30, I-50, and I-100. Red and black dotted lines indicate pyridinium salt (1635 cm^{-1}) and PP (1375 cm^{-1}) peaks, respectively. (c) Ratio of pyridinium salt (1635 cm^{-1}) to PP (1375 cm^{-1}) absorbances for each specimen. Data are presented as the means ± SDs measured from 10 different specimens. (d) Surface-coated pyridinium group density (mmol cm^{-2}) measured by the fluorescein staining method.[2] Three independent specimens were examined. (e) Correlation between FT-IR absorbance ratio (1635 cm^{-1}/1375 cm^{-1}) and pyridinium group density (r = 0.98).

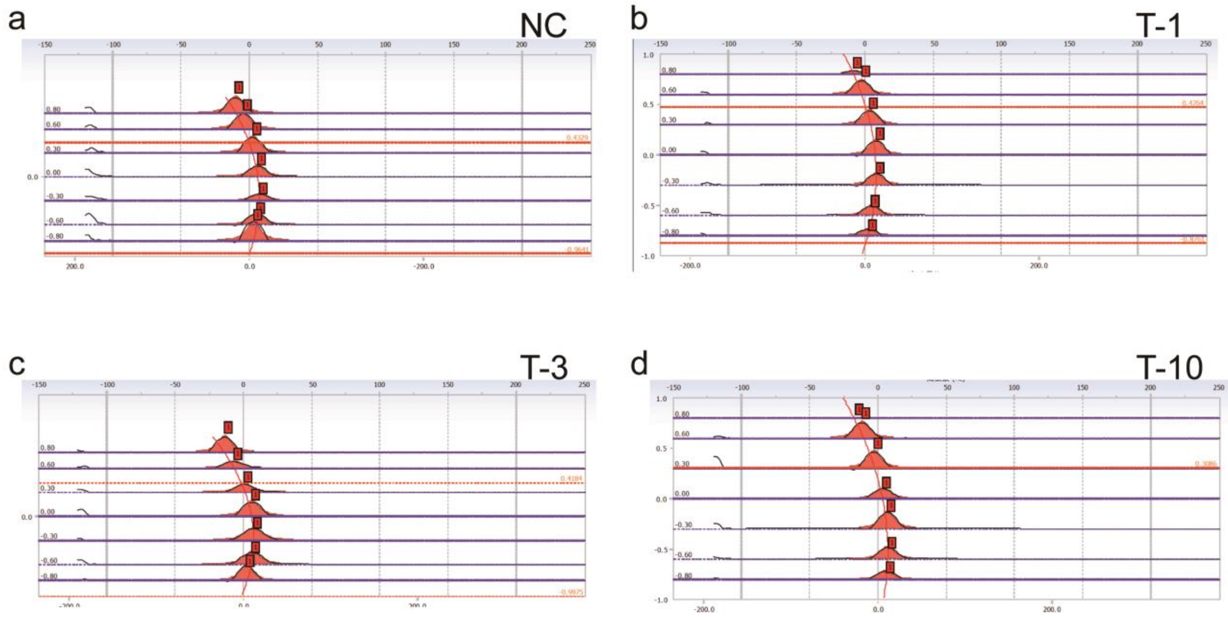

Figure S7. Electro-osmosis profiles of (a) NC, (b) T-1, (c) T-3, and (d) T-10. The apparent electrophoretic mobility of the monitored particles was measured at seven different points in the cell's depth direction. Each electro-osmosis profile was used to determine the velocity of the electro-osmotic flow at the solid interface and derive the zeta potential of the flat surface.

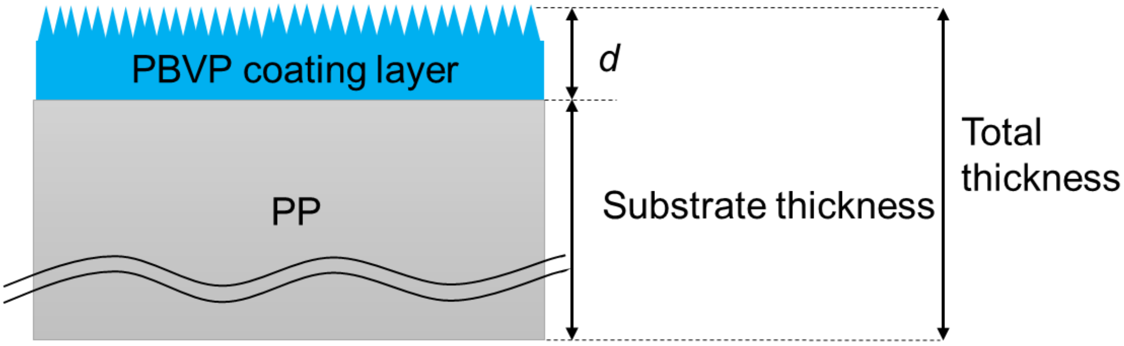

Figure S8. Schematic cross-sectional view of PBVP-coated PP. Coating thickness (*d*) refers to the thickness of the PBVP coating layer, and total thickness is the thickness of the entire specimen.

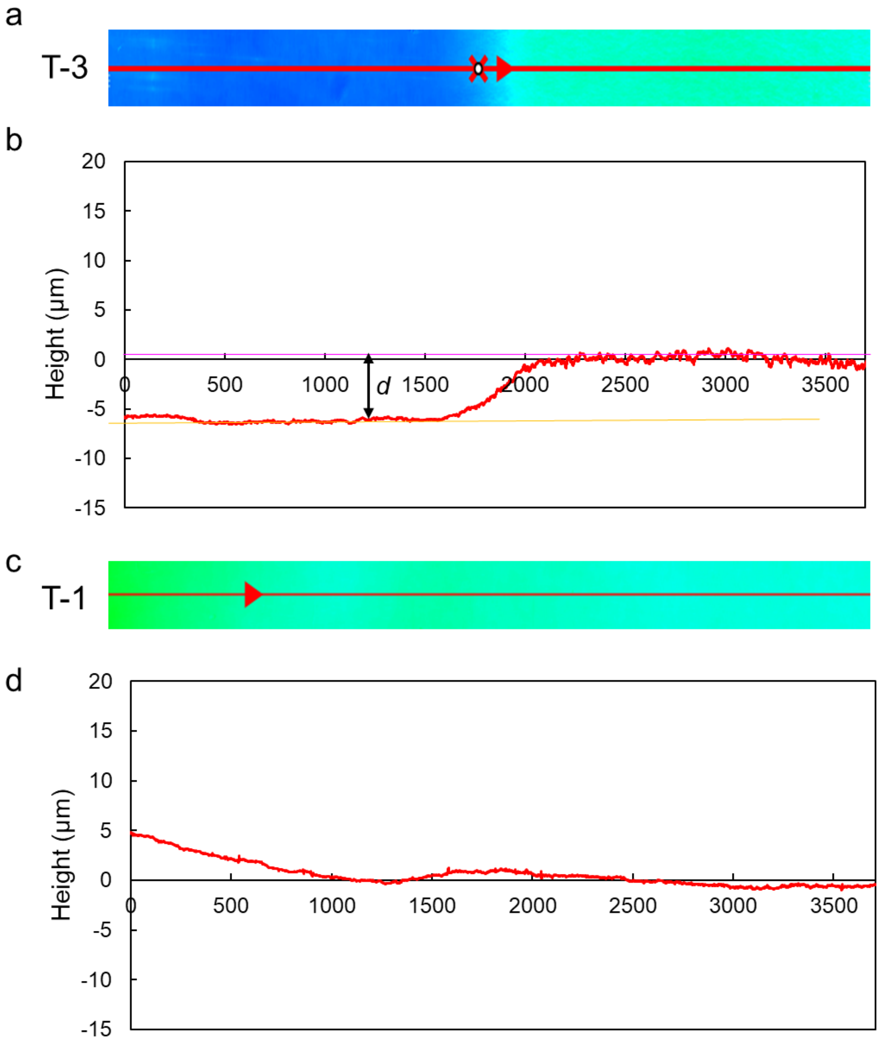

Figure S9. (a) 2D image of the boundary between the non-coated and PBVP-coated areas of T-3. The red line shows the line scan position. (b) Surface topography of the area line-scanned in (a). The horizontal axis displays the *x*-coordinate, while the vertical axis displays the height. The flat orange line indicates the non-coated area, while the pink line shows the unevenness in the coated area. The difference in height between these two lines was used to calculate *d*. (c) 2D image of T-1. (d) Surface topography of the area line-scanned in (c). The *d* value of T-1 could not be calculated because the border between coated and noncoated areas could not be detected.

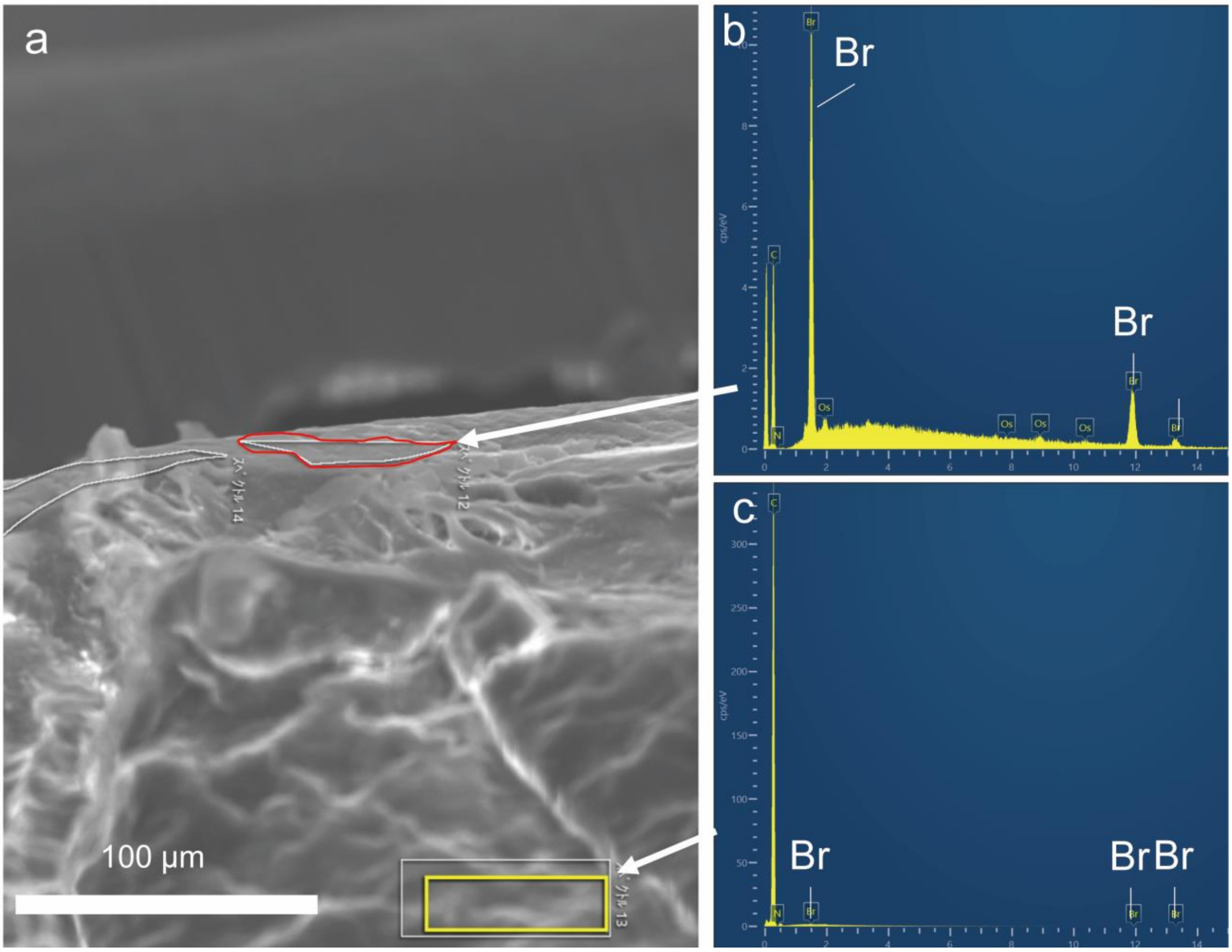

Figure S10. (a) Cross-sectional SEM image of PBVP-coated PP. (b) EDX spectra of areas enclosed by (b) red and (c) yellow lines.

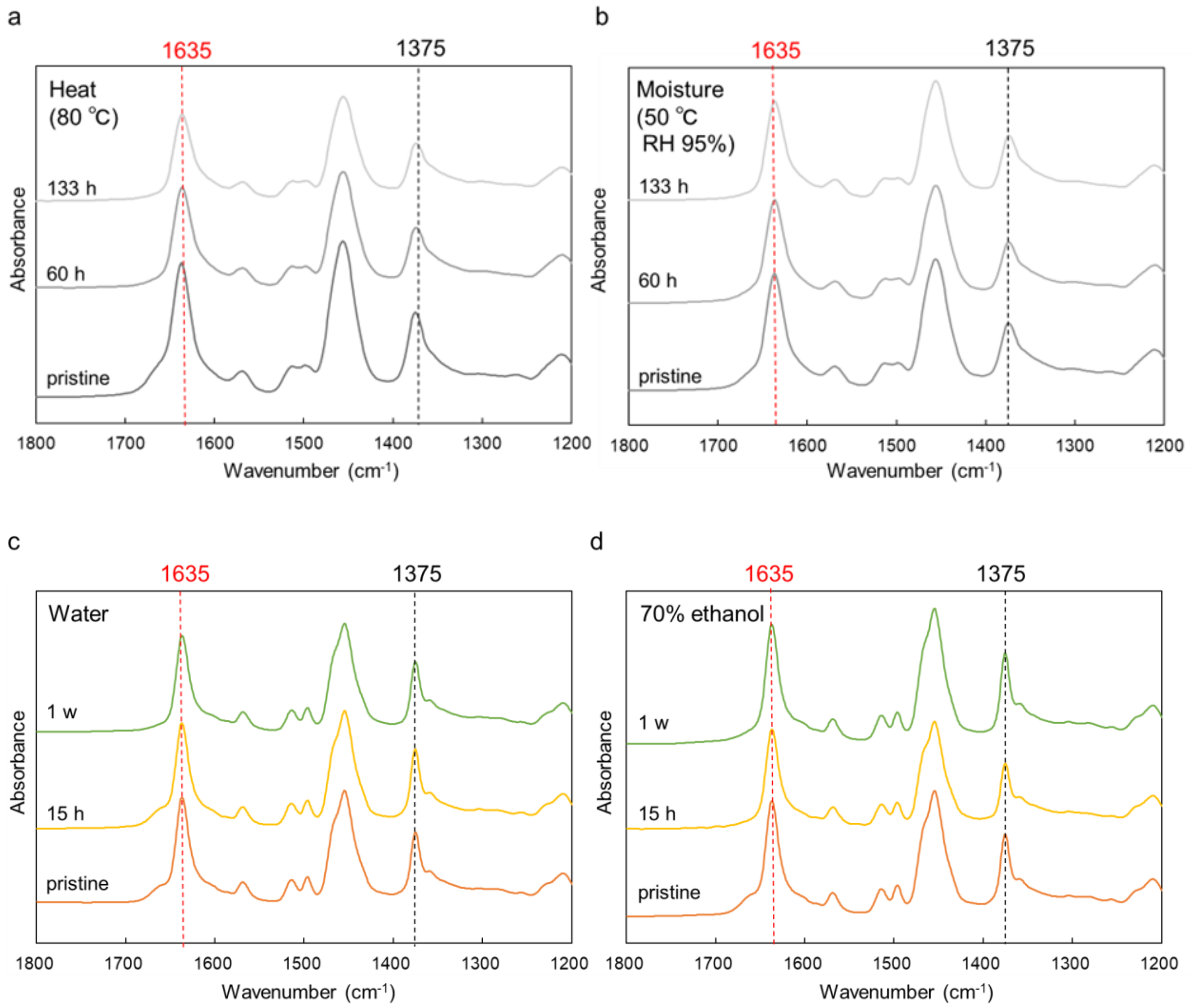

Figure S11. FT-IR spectra after the (a) heat and (b) moisture treatments of T-10 for 60 and 133 h and after immersion in (c) water and (d) 70% ethanol for 15 h and 1 week. Red and black dotted lines indicate pyridinium salt (1635 cm^{-1}) and PP (1375 cm^{-1}) peaks, respectively.

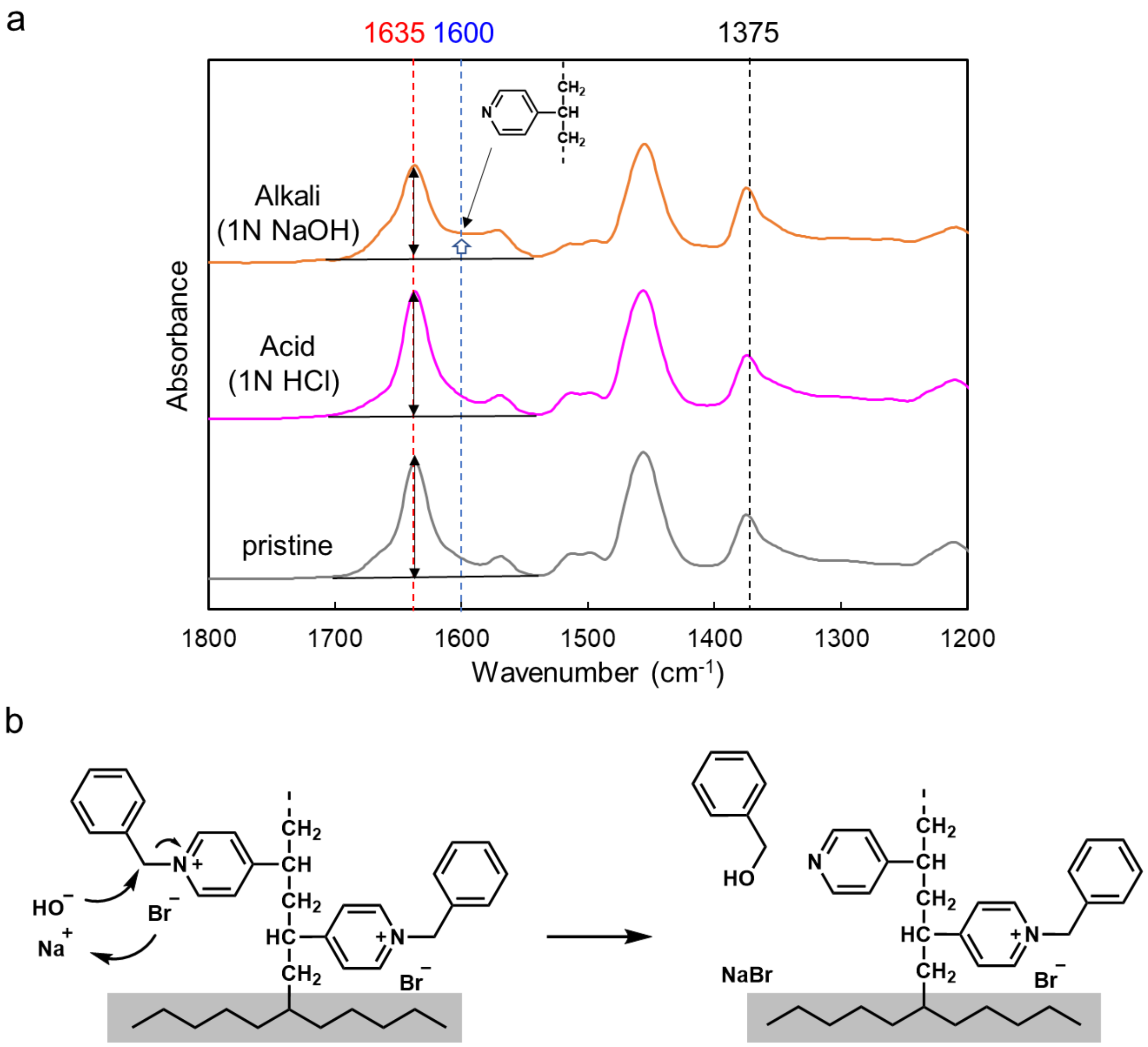

Figure S12. (a) FT-IR spectra after alkali (1 N NaOH) and acid (1 N HCl) treatments. The solid black line indicates the baseline for measuring absorbance at 1635 cm^{-1}. Red, blue. and black dotted lines indicate pyridinium salt (1635 cm^{-1}), pyridine (1600 cm^{-1}), and PP (1375 cm^{-1}) peaks, respectively. (b) Schematic diagram of hypothesized changes in PBVP-coated PP after alkali treatment.

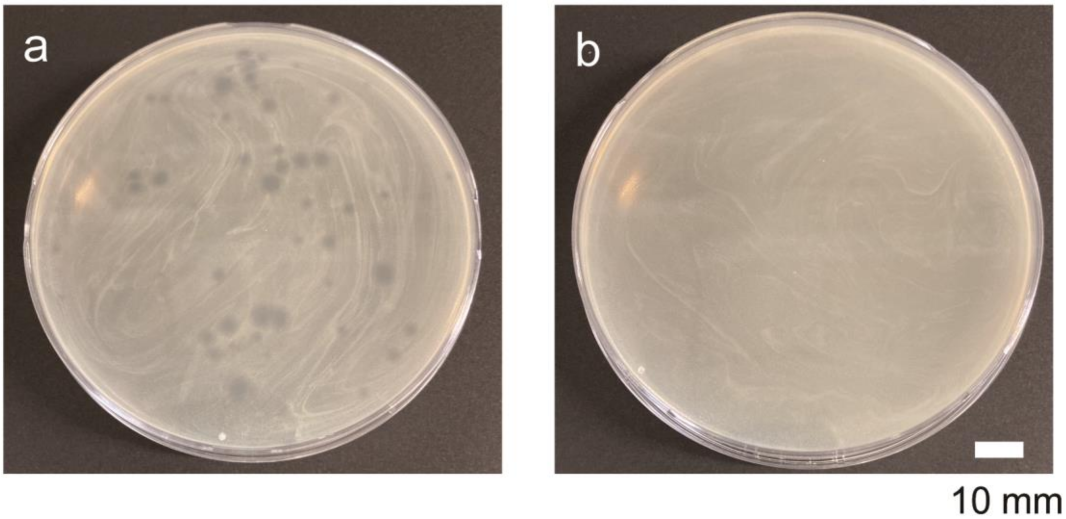

Figure S13. Plate of plaque assay with bacteriophage φ6 in contact with (a) NC and (b) T-10 for 60 min.

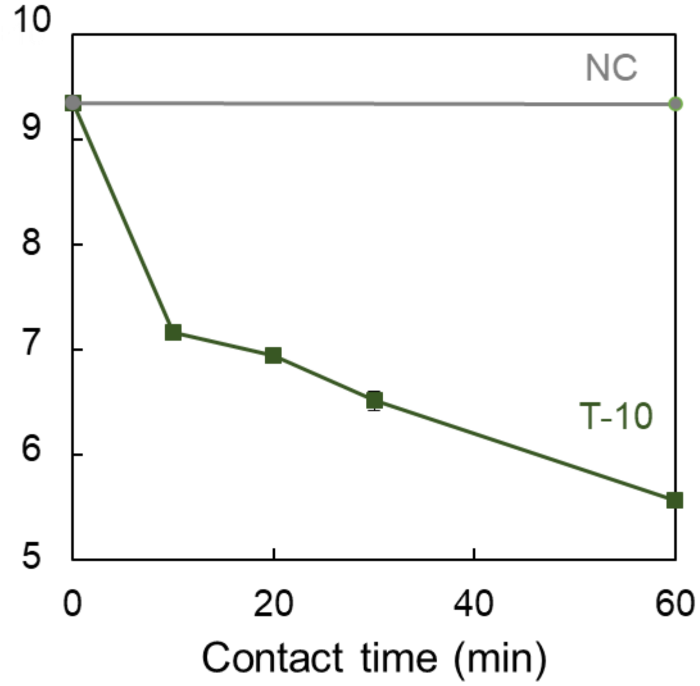

Figure S14. Reverse transcription-quantitative polymerase chain reaction (RT-qPCR) analysis for bacteriophage φ6 S_1 gene. All examinations were performed in triplicate and independently. The results are presented as the corresponding means ± SDs. RT-qPCR was performed using Taqman fast Virus 1-step master Mix (Thermo Fisher Scientific, Waltham, MA) using QuantStudio 3 (Thermo Fisher Scientific, Waltham, MA) and the following set of primers/probes.

Forward primer, 5'- TGGCGGCGGTCAAGAGC -3';

Reverse primer, 5'-GGATGATTCTCCAGAAGCTGCTG -3';

Probe, 5'-(FAM) CGGTCGTCGCAGGTCTGACACTCGC (TAMRA) -3'.[3]

The cycle threshold (Ct) values of RT-qPCR were converted into RNA copy numbers based on a standard curve prepared from known copy numbers of bacteriophage φ6 RNA.

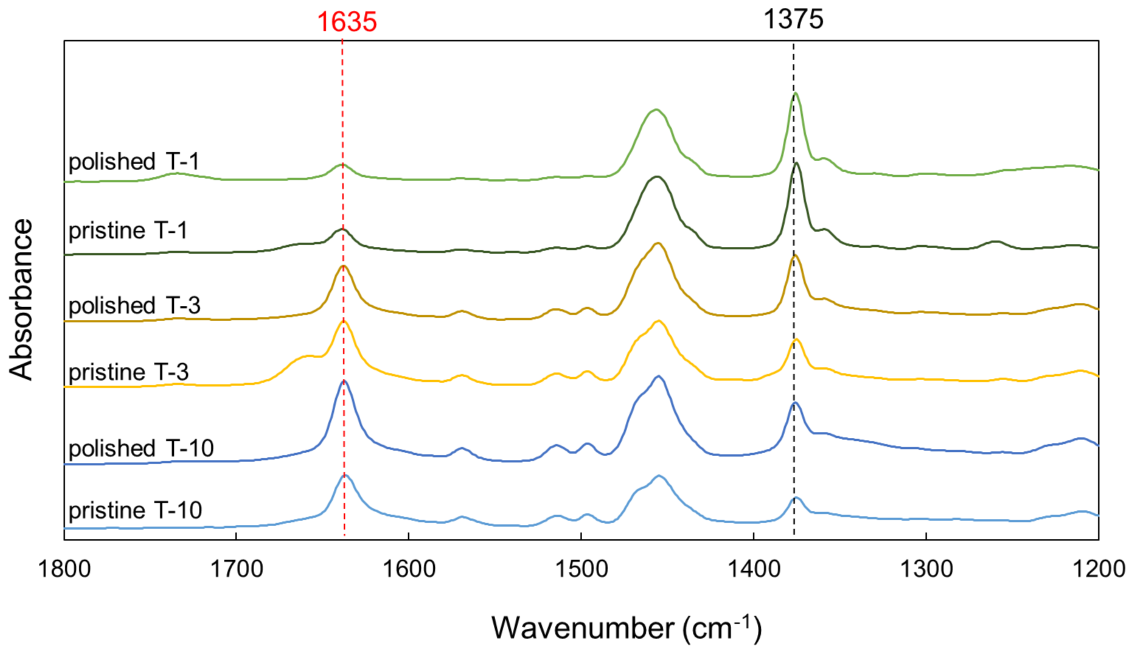

Figure S15. FT-IR spectra of polished T-1, T-3, and T-10. Red and black dotted lines indicate pyridinium salt (1635 cm^{-1}) and PP (1375 cm^{-1}) peaks, respectively.

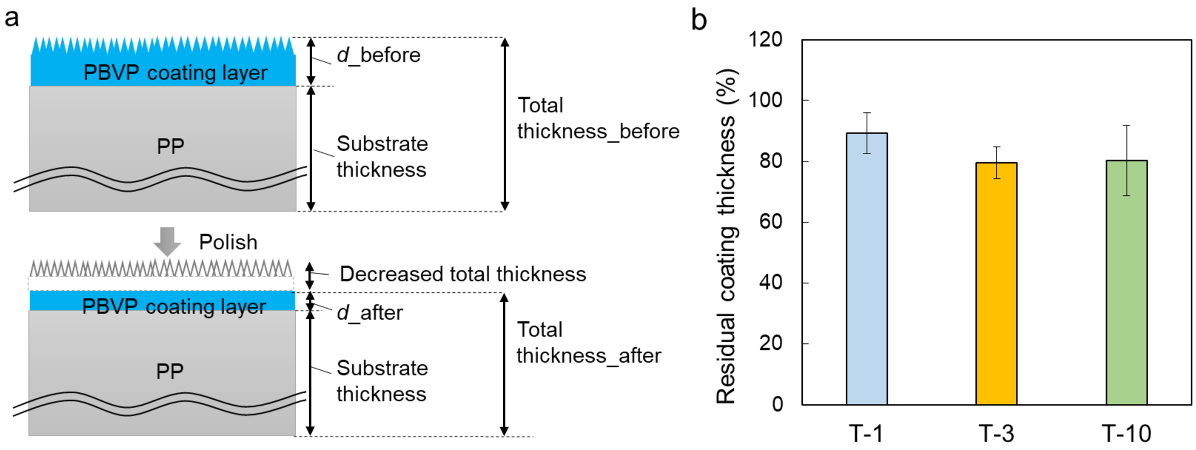

Figure S16. (a) Schematic cross-sectional view of PBVP-coated PP before and after polishing. The thickness of the entire specimen (total thickness) was measured with a digital micrometer before and after polishing, and the difference due to polishing (decreased total thickness) is shown in Figure 8a. The thickness of four NC samples were measured, and the mean was used as the substrate thickness. Coating thickness (*d*) was calculated by subtracting substrate thickness from total thickness. (b) Residual coating thicknesses of T-1, T-3, and T-10 determined from the coating thicknesses calculated before and after polishing in (a). Four pieces were polished for each sample, and the results were expressed as the corresponding means ± SDs.

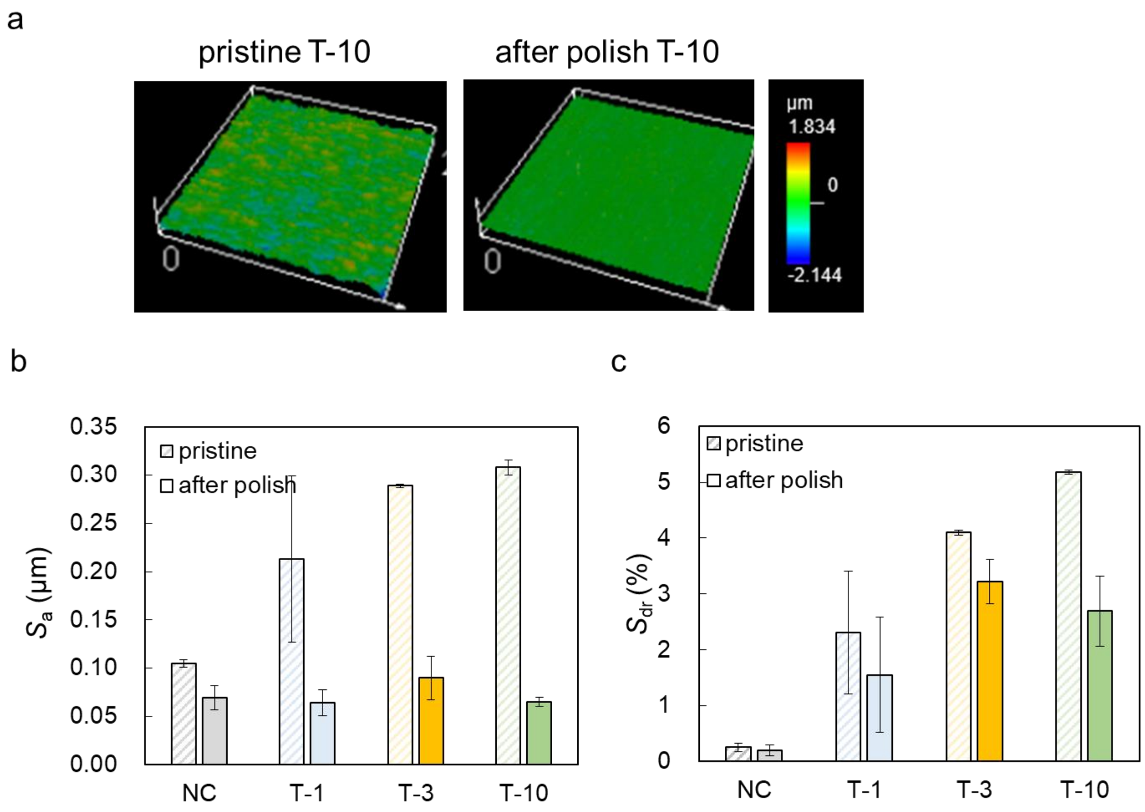

Figure S17. (a) 3D surface image of polished T-10 acquired using 3D CLSM (50 × objective, ~250 μm × 250 μm). (b) Average surface roughness (S_a) and (c) interfacial expansion area ratio (S_{dr}) values of polished T-1, T-3, and T-10 calculated using 3D CLSM. All data are represented as means ± SDs.

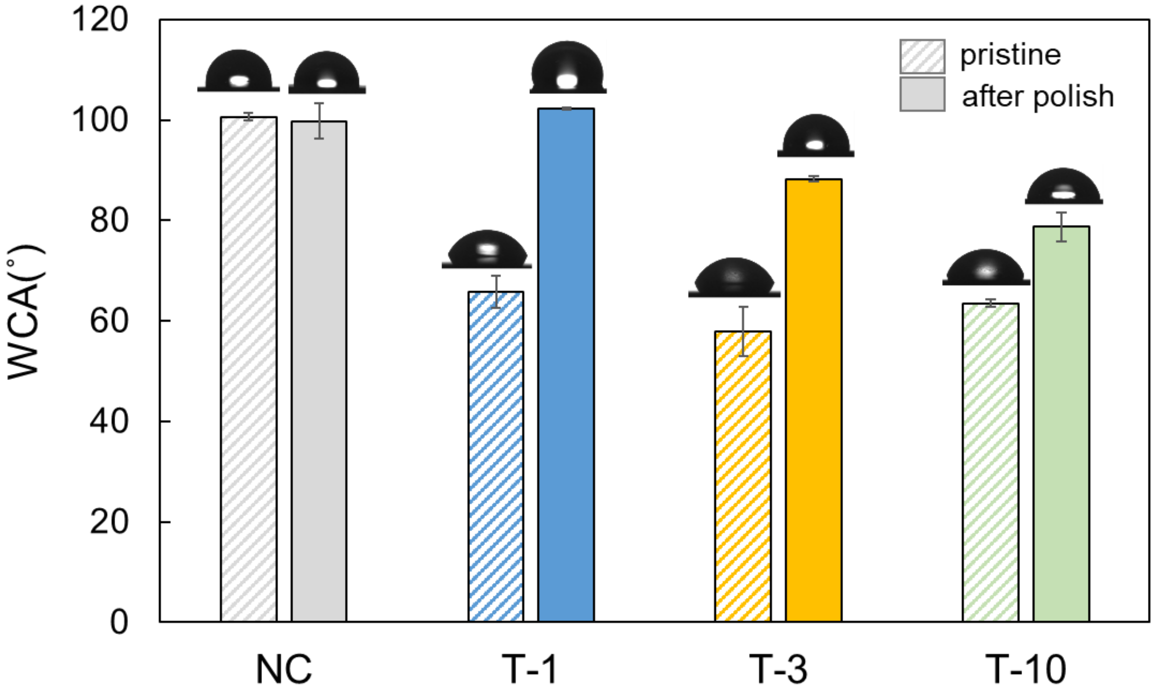

Figure S18. Water contact angles (WCAs) of 2 μL water droplets on polished T-1, T-3, and T-10. Data represent the means and SDs from five different spots on the same sample.

REFERENCES

(1) Ajitha A. R; Arif P, M.; M. K, A.; Mathew, L. P.; V. G, G.; Kalarikkal, N.; Thomas, S.; Volova, T. An Effective EMI Shielding Material Based on Poly(trimethylene terephthalate) Blend Nanocomposites with Multiwalled Carbon Nanotubes., *New Journal of Chemistry* **2018**, *42* (16), 13915–13926.

(2) Dong, J. J.; Muszanska, A.; Xiang, F.; Falkenberg, R.; van de Belt-Gritter, B.; Loontjens, T. Contact Killing of Gram-Positive and Gram-Negative Bacteria on PDMS Provided with Immobilized Hyperbranched Antibacterial Coatings., *Langmuir* **2019**, *35* (43), 14108–14116.

(3) Gendron, L.; Verreault, D.; Veillette, M.; Moineau, S.; Duchaine, C. Evaluation of Filters for the Sampling and Quantification of RNA Phage Aerosols., *Aerosol Science and Technology* **2010**, *44* (10), 893–901.
